# Reversible protein affinity-labelling using bromomaleimide-based reagents†
†Electronic supplementary information (ESI) available: ^1^H and ^13^C spectra for all new and known compounds, and ES-MS spectra for all reactions with proteins described herein. See DOI: 10.1039/c3ob40239hClick here for additional data file.



**DOI:** 10.1039/c3ob40239h

**Published:** 2013-03-05

**Authors:** Ramiz I. Nathani, Vijay Chudasama, Chris P. Ryan, Paul R. Moody, Rachel E. Morgan, Richard J. Fitzmaurice, Mark E. B. Smith, James R. Baker, Stephen Caddick

**Affiliations:** a Department of Chemistry , University College London , 20 Gordon Street , London , WC1H OAJ , UK . Email: VPEnterprise@ucl.ac.uk ; Fax: +44 (0)20 7679 7463 ; Tel: +44(0)20 3108 5071

## Abstract

Reversible protein biotinylation is readily affected *via* conjugation with a bromomaleimide-based reagent followed by reductive cleavage. The intermediate biotinylated protein constructs are stable at physiological temperature and pH 8.0. Quantitative reversibility is elegantly delivered under mild conditions of using a stoichiometric amount of a bis-thiol, thus providing an approach that will be of general interest in chemical biology and proteomics.

## Abstract 2

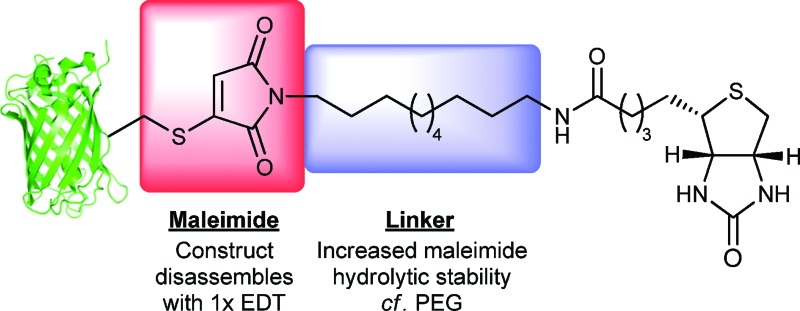
A mild and highly efficient, reversible protein biotinylation method using a hydrolytically stable linker and mild disassembly conditions is described.

To facilitate the study of a plethora of complex biological processes, robust methods for the selective enrichment of tagged proteins from complex biological mixtures is of primary importance. Use of the biotin–avidin interaction, to facilitate biotin-labeled protein enrichment or immobilisation, is a well-established technique.^1–3^ However, classical approaches based on this method are limited in that in order to ultimately retrieve the protein of interest the very strong biotin–avidin interaction (*K*_a_ = 1.7 × 10^15^ M^–1^) must be disrupted. This is often achieved under harsh, denaturing conditions that are not experimentally useful.^1^ Strategies have been developed to try and address this problem. The first approach requires the design of analogues of biotin which bind with lower affinity to avidin.^4^ Although useful, this approach is inherently hampered as increased ease of elution comes at the price of weaker initial binding. A second approach is to design affinity probes which maintain the native biotin–avidin interaction but which incorporate a cleavable linker.^1,2^

We have recently reported a new approach to cysteine bioconjugation through the use of bromomaleimides and bromopyridazinediones.^5^ To date, our approach has provided access to complex bioconjugates in high yields, without prior activation of reagents. As part of this programme, we reported a new bromomaleimide-based affinity-tagging reagent which allows for the facile and reversible biotinylation of proteins.^5*c*^ A buffered solution of a single point mutant (L111C) of the SH2 domain of the Grb2 adapter protein was affinity-tagged, and incubated, prior to cleavage in a large excess of *beta*-mercaptoethanol (BME, 100 equivalents, 25 mM). Although we have found this protocol to be useful, we discovered that prolonged incubation of the affinity-tagged protein lead to some unwanted maleimide hydrolysis (*ca.* 40% hydrolysis at 37 °C after 4 h at pH 8.0) which rendered the labeling, at least partially, irreversible, resulting in a sub-optimal yield of recovered protein. Furthermore, the relatively harsh cleavage conditions are unlikely to be compatible with proteins that contain sensitive disulfide bonds, potentially resulting in unfavourable protein unfolding, aggregation or disulfide scrambling. This limitation is particularly important when one considers that disulfide bonds are often very important for the structural stability of proteins.^6^

We report herein a novel affinity tag reagent, which yields bioconjugates that are stable to maleimide hydrolysis over extended periods of time at physiological temperature and pH 8.0. Furthermore, we describe the subsequent liberation of native protein from the bioconjugate under mild conditions, a single equivalent of a dithiol, in both a model system and in proof of concept biotin–streptavidin pull-down experiments.

The nature of the *N*-substituent of a maleimide is known to effect the rate of hydrolysis to the corresponding maleamic acid.^7^ Indeed, we have recently described the exploitation of this effect for the irreversible labelling of proteins with hydrolytically unstable maleimides bearing electron withdrawing groups on nitrogen, such as *N*-phenylmaleimides.^5*c*^ Given that electron withdrawing substituents on the maleimide nitrogen promote hydrolysis,^5c,7^ we envisaged that incorporation of an electron donating substituent on the maleimide nitrogen would suppress such an effect.

Thus, *n*-butyl and methoxy substituents (inductively and mesomerically electron donating groups, respectively), were incorporated into bromomaleimides to form compounds **1** and **2** for model hydrolysis studies. We also synthesised ethylglycol methyl ether bromomaleimide **3** to investigate whether the hydrolysis of our previously reported PEG-biotin linker was intimately linked to the ethyl glycol ether functionality. Cyclohexylmethyl bromomaleimide **4** was also synthesised to assess whether a *N*-cyclohexylmethyl group, as in the case reported by Hermanson,^8^ would reduce hydrolysis in this system. Bromomaleimides **1–4** were readily synthesised by refluxing bromomaleic anhydride with the corresponding amine in acetic acid for 3 h. The crude reaction was purified by column chromatography to yield pure bromomaleimides **1–4** with yields ranging from 54% to 72% (see ESI† for details).

Single point mutant (L111C) of the SH2 domain of Grb2 adaptor protein, **5**, which otherwise does not contain cysteine residues, was chosen as a model protein. This protein was treated with one equivalent of bromomaleimide reagent for 1 h at 0 °C (these bioconjugation reactions may also be performed at room temperature or 37 °C). Analysis of the crude reaction mixtures by LCMS indicated that, despite varying the *N*-substituent, reactions with bromomaleimides **1–4** proceeded with quantitative conversion. The hydrolytic stability of the conjugates was then assessed by incubation at 37 °C for 4 h at pH 8.0 (Table 1). Although the conjugates of bromomaleimides **2** and **3** showed significant hydrolysis, the conjugates of **1** and **4** were completely stable. On this basis, it was concluded that a non-functionalised alkyl chain (*i.e.* an inductively electron donating group) on the maleimide nitrogen should be sufficient to minimise hydrolysis.

**Table 1:** Reaction of Grb2 SH2 (L111C) **5** with bromomaleimides **1–4** and stability of the formed conjugates at 37 °C for 4 h at pH 8.0.^*a*^

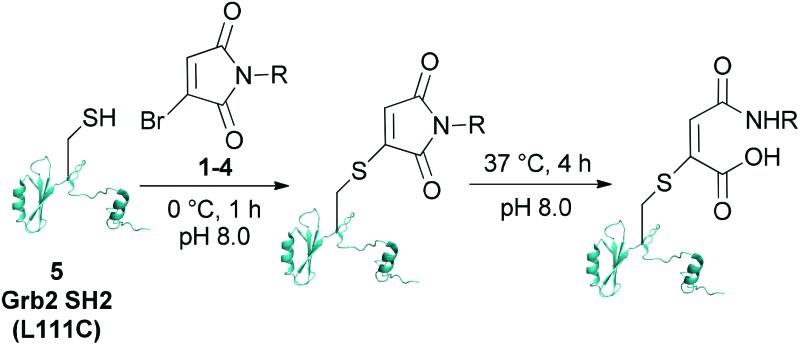			
Maleimide	R group	Conversion of Grb2 SH2 (L111C) **5**	Hydrolysis of conjugate at 37 °C for 4 h
**1**	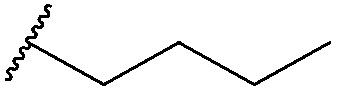	>99%	<1%
**2**	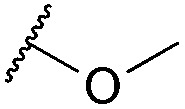	>99%	40%
**3**	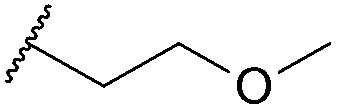	>99%	45%
**4**	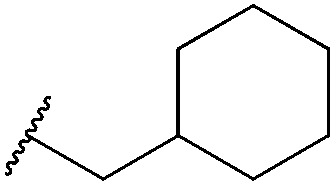	>99%	<1%

^*a*^Detailed experimental details are given in the ESI.

In light of the results with bromomaleimides **1** and **4**, a biotinylated-bromomaleimide derivative with a non-functionalised alkyl chain, dodecane-biotin bromomaleimide **6**, was synthesized and its hydrolytic stability evaluated. Decane-1,10-diamine, rather than butyl-1,4-diamine or 4-(aminomethyl)cyclohexanamine, was chosen as the spacer to minimise any interference between the protein and the biotin–streptavidin interaction. Bromomaleimide **6** was synthesized by coupling mono Boc protected decane-1,10-diamine with activated biotin, followed by TFA deprotection of the Boc group and reaction with bromomaleic anhydride in acetic acid (see ESI† for details).

The reactivity of biotinylated bromomaleimide **6** with Grb2 SH2 (L111C) **5** was tested and, as anticipated, reaction at 0 °C for 1 h resulted in the formation of bioconjugate **8** in quantitative conversion (Scheme 1). In order to evaluate whether the efficiency of bioconjugation with bromomaleimide **6** was specific to Grb2 SH2 (L111C) **5**, reaction was also carried out with GFP (S147C) **7**.^5*b*^ Use of a fluorescent protein would also provide a parameter by which to quantify the release of protein in our reversible biotinylation methodology (*vide infra*). Gratifyingly, bioconjugation with GFP (S147C) **7** also proceeded efficiently, forming bioconjugate **9** in quantitative conversion (Scheme 1). Conjugates **8** and **9** were then tested for stability by heating them at 37 °C, pH 8.0 for 4 h. Most pleasingly, as observed with the *n*-butyl and cyclohexylmethyl model systems, no hydrolysis was observed.‡
‡Analogous results were observed for 4 h at 37 °C, pH 7.4.


**Scheme 1:**
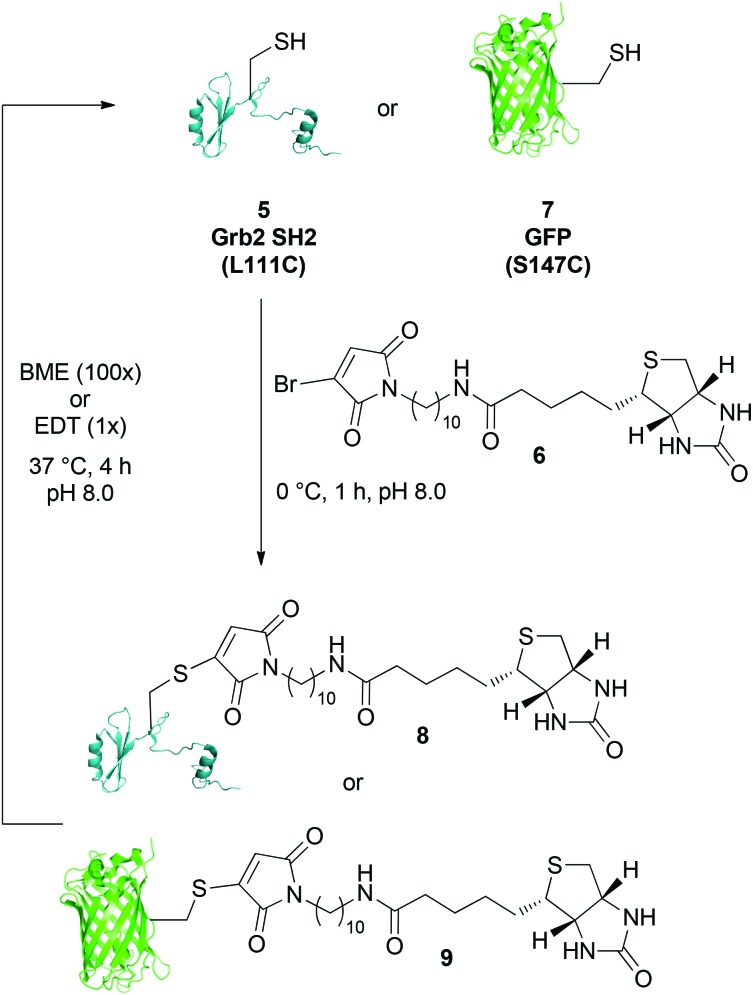
Reaction of dodecane-biotin bromomaleimide **6** with Grb2 SH2 (L111C) **5** and GFP (S147C) **7**, and release of the free proteins using BME (100×) or EDT (1×).

Having established that adducts **8** and **9** are stable to hydrolysis at physiological temperature at pH 8.0 over a prolonged period of time, and in view of our goal of providing a general protocol for the reversible biotinylation of proteins, we turned our attention to developing a mild set of cleavage conditions for the liberation of a protein of interest from the affinity label. In our previous report, we described that Grb2 SH2 (L111C) maleimide conjugates react with a single equivalent of thiol to form bisthioethers.^5a,c^ In an environment of an excess of thiol, however, these bioconjugate thioethers are unstable, indicating that the process is under equilibrium (Scheme 2(a)). To date, this reversibility has been exploited by adding a large excess of thiol (*i.e.* 100× of BME or Glutathione) to facilitate the release of free protein. However, a major disadvantage of this technique is that it requires a large excess of thiol, which, as discussed above, could limit the applicability of this approach.

**Scheme 2:**
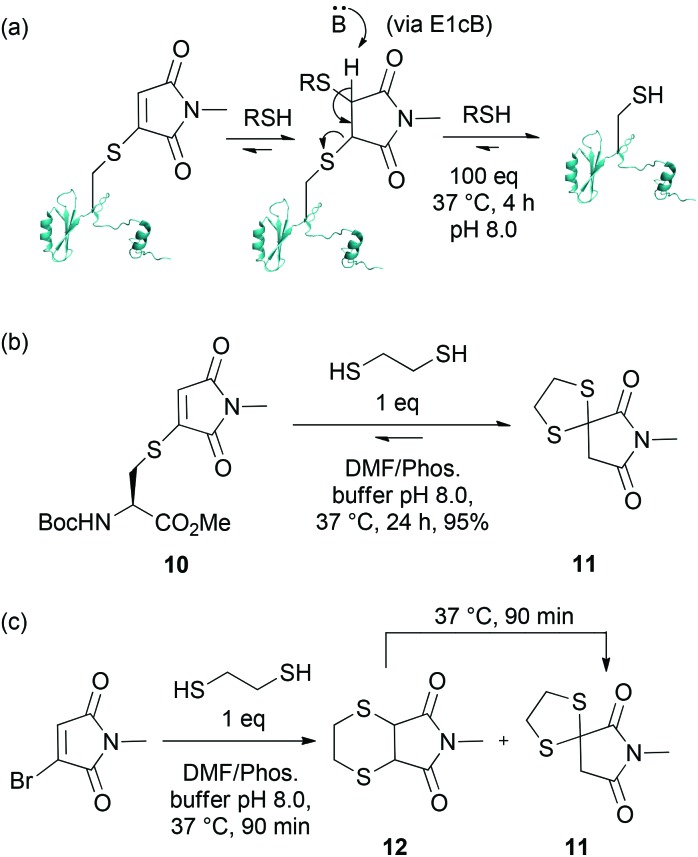
(a) Reaction of Grb2 SH2 (L111C) maleimide bioconjugate with an excess of thiol; (b) Reaction of cysteine–maleimide conjugate with EDT; (c) Reaction of *N*-methylbromomaleimide with EDT, and rearrangement of **12** to **11**.

To improve upon our capture and release methodology, it was essential to facilitate the release of free protein without using an excess of thiol (Scheme 2(a)). We rationalised that dithiosuccinimides are unlikely to be stable because of the low p*K*_a_ of the succinimide proton – and that this is key for the reversibility of this system. We envisaged that a tether-type approach using bidentate thiol nucleophiles which could form bi- or spirocyclic adducts by trapping the reactive dithiosuccinimides into a stable, unreactive adduct, would liberate free protein without the need for an excess of thiol. Thus, we embarked upon a model study using thiomaleimide **10**. Pleasingly, reaction of **10** with a single equivalent of 1,2-ethanedithiol (EDT) afforded spirocyclic dithiane **11** in 95% yield after 24 h (Scheme 2(b)). Further insight into the mechanism of cleavage of thiomaleimide with ethanedithiol was provided by treatment of *N*-methylbromomaleimide with EDT, which revealed the formation of two major products, spirocyclic dithiane **11** and bicyclic bisthioether **12** in a 65 : 35 ratio after 90 min at 37 °C (Scheme 2(c)). However, after prolonged incubation at 37 °C, bicyclic bisthioether **12** was completely converted to the thermodynamic product, dithiane adduct **11**.

The EDT cleavage strategy was evaluated by incubation of biotin–bromomaleimide conjugates **8** and **9** with a single equivalent of EDT for 4 h at 37 °C, pH 8.0. Analysis of the crude reaction mixtures by LCMS revealed complete regeneration of free unmodified proteins with no evidence for the significant formation of side-products (Scheme 1).‡ As a control, our previously reported cleavage conditions were also applied to biotinylated conjugates **8** and **9**.^5*c*^ Although reaction of **9** with excess BME (100 equivalents) for 4 h at 37 °C, pH 8.0 yielded GFP (S147C) **7** in quantitative conversion, sub-optimal yield (*ca.* 90%) was observed for release of Grb2 SH2 (L111C) **5** from adduct **8** as a result of the persistence of succinimide formed from mono-addition of BME to **8**; thus highlighting a further advantage of our novel dithiol cleavage strategy.

Having identified an affinity tag which is stable to hydrolysis at 37 °C for several hours and a reagent which enables clean release of protein with only a single equivalent of reducing thiol (EDT), we sought to evaluate these optimised protocols in proof of concept pull down experiments (Scheme 3).

**Scheme 3:**
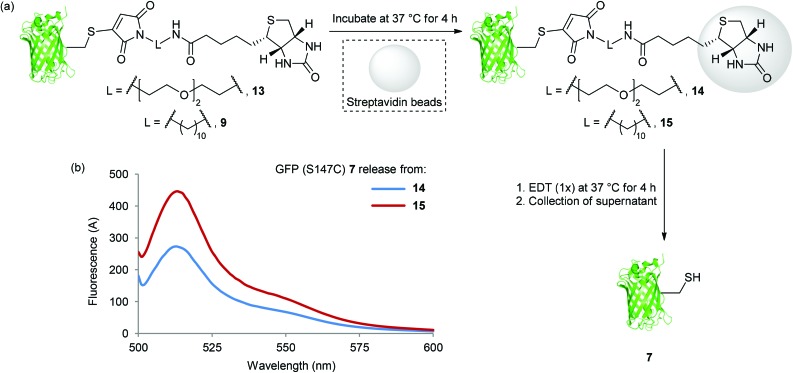
(a) Binding of **13** and **9** to Streptavidin beads, and release of GFP (S147C) **7** with EDT (1×); (b) Fluorescence spectra of GFP (S147C) **7** released from bead washes after incubation of **14** and **15** with EDT (1×) at 37 °C for 4 h.

This also provided an opportunity to compare our previously reported PEG maleimide reagent^5*c*^ with our new decane bromomaleimide reagent **6**. Thus, GFP (S147C) **7** was modified with PEG and decane maleimide reagents at 0 °C for 1 h, forming bioconjugates **13** and **9** in quantitative conversion. Bioconjugates **13** and **9** were then incubated directly with streptavidin beads at 37 °C for 4 h (Scheme 3(a)). In each case, after the incubation period, unbound GFP-bioconjugate was removed by washing the beads with PBS buffer solution (3 × 10-fold dilution). Incubation of the washed GFP (S147C) loaded streptavidin beads (**14** and **15**) with EDT at 37 °C for 4 h resulted in release of GFP (S147C) **7** (Scheme 3(a)); this was determined by fluorescence analysis (*vide supra*) of the supernatant (Scheme 3(b)). Gratifyingly, a significantly increased level of release from the decane conjugate **9** was observed, likely due to superior hydrolytic stability. This clearly demonstrates a substantial advantage of **6** over our previously reported PEG linked affinity tag reagent and the applicability of our mild cleavage conditions to reversible protein affinity labelling using streptavidin beads; thus highlighting the potential of our optimised cleavable tag/EDT strategy.

## Conclusions

The continuing study of the interaction of proteins with other biomolecules is vital in furthering our understanding of human physiology. The development of proteomic technologies will facilitate the discovery of new diagnostics and drugs that will underpin 21st century medicine.^9,10^ Key to this is the development of novel conjugation technologies that allow for the enrichment, manipulation and analysis of proteins using mild conditions that are compatible with maintaining native protein structure and function.^11^ We have described an elegant approach that enables the reversible affinity-labelling of proteins. We envisage that this technology will have applications in a variety of affinity applications but particularly in proteomic applications where the capture, release and analysis of native proteins and their complexes is highly desirable.

The authors are grateful to Dr D. Papaioannou and Prof. G. Waksman for donation of the Grb2 SH2 plasmid. We also gratefully acknowledge the EPSRC, BBSRC, Wellcome Trust, UCL, UCLB and GSK for support of our programme.
