# Supplementary material for: Reversible protein affinity-labelling using bromomaleimide-based reagents
Source: Org Biomol Chem. 2013 Mar 5;11(15):2408–11. doi: 10.1039/c3ob40239h (PMC3763775; doi:10.1039/c3ob40239h)
Supplement: Supplementary file 1 [file OB-011-c3ob40239h-s001.pdf]

This journal is © The Royal Society of Chemistry 2013

Reversible protein affinity-labelling using bromomaleimide-based reagents

Ramiz I. Nathani, Vijay Chudasama, Chris P. Ryan, Paul R. Moody, Rachel E. Morgan, Richard J. Fitzmaurice, Mark E. B. Smith, James R. Baker and Stephen Caddick*

Department of Chemistry, University College London, 20 Gordon Street, London, WC1H OAJ, UK

Tel: +44 (0)20 3108 5071; Fax: +44 (0)20 7679 7463; E-mail: VPEnterprise@ucl.ac.uk

General Experimental

All reagents were purchased from Aldrich or AlfaAesar and were used as received without further purification. Where described below petrol refers to petroleum ether (40-60 °C). All reactions were monitored by thin-layer chromatography (TLC) on pre-coated SIL G/UV254 silica gel plates (254 μm) purchased from VWR. Flash column chromatography was carried out with Kiesegel 60M 0.04/0.063mm (200-400 mesh) silica gel. ^{1}H and ^{13}C NMR spectra were recorded at ambient temperature on a Bruker Avance 400 instrument operating at a frequency of 400 MHz for ^{1}H and 100 MHz for ^{13}C, a Bruker Avance 500 instrument operating at a frequency of 500 MHz for ^{1}H and 125 MHz for ^{13}C, and a Bruker Avance 600 instrument operating at a frequency of 600 MHz for ^{1}H and 150 MHz for ^{13}C in $CDCl_3$ or CD_3OD (as indicated below). The chemical shifts (δ) for ^{1}H and ^{13}C are quoted relative to residual signals of the solvent on the ppm scale. ^{1}H NMR peaks are reported as singlet (s), doublet (d), triplet (t), quartet (q), quintet (quintet), broad (br) or multiplet (m). Coupling constants (*J* values) are reported in Hertz (Hz) and are H-H coupling constants unless otherwise stated. Signal multiplicities in ^{13}C NMR were determined using the distortionless enhancement by phase transfer (DEPT) spectral editing technique. Infrared spectra were obtained on a Perkin Elmer Spectrum 100 FTIR Spectrometer operating in ATR mode with frequencies given in reciprocal centimetres (cm^{-1}). Melting points were measured with a Gallenkamp apparatus and are uncorrected. Optical rotations were measured using a Perkin Elmer 343 polarimeter. Mass spectra were obtained on a VG70-SE mass spectrometer.

LC-MS was performed on protein samples using a Waters Acquity uPLC connected to Waters Acquity Single Quad Detector (SQD). Column: Acquity uPLC BEH C18 1.7μm 2.1 x 50 mm. Wavelength: 254 nm. Mobile Phase: 95:5 Water (0.1% Formic Acid): MeCN (0.1% Formic Acid). Gradient over 4 min (to 5:95 Water (0.1% Formic Acid): MeCN (0.1% Formic Acid). Flow Rate: 0.6 mL/min. MS Mode: ES^{+}. Scan Range: m/z = 85-2000. Scan time: 0.25 sec. Data obtained in continuum mode. The electrospray source of the MS was operated with a capillary voltage of 3.5 kV and a cone voltage of 50 V. Nitrogen was used as the nebulizer and desolvation gas at a total flow of 600 L/h. Total mass spectra for protein samples were reconstructed from the ion series using the MaxEnt 1 algorithm pre-installed on MassLynx software.

General procedure for the synthesis of bromomaleimide derivatives 1-4

To a solution of amine (1 mmol) in AcOH (3 mL) was added 3-bromo-maleic anhydride (1 mmol) and the reaction mixture was heated under reflux for 3 h. After this time, the reaction mixture was

cooled to room temperature, toluene was added (3 mL), the solvents were removed *in vacuo* and the crude residue was purified as described below.

3-Bromo-1-(butyl)-pyrrole-2,5-dione 1

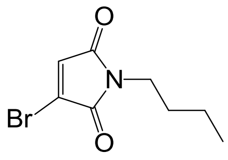

Purified by column chromatography (5% to 50% EtOAc/Petrol) gave 3-bromo-1-(butyl)-pyrrole-2,5-dione **1** as a yellow solid (166 mg, 0.72 mmol, 72%): ^{1}H NMR (400 MHz, CD_3OD) δ 7.11 (s, 1H), 3.55 (t, J = 7.0 Hz, 2H), 1.62-1.54 (m, 2H), 1.37-1.28 (m, 2H), 0.95 (t, J = 7.0 Hz, 2H); ^{13}C NMR (100 MHz, CD_3OD) δ 168.8 (C), 165.7 (C), 132.1 (C), 130.5 (CH), 38.0 (CH_2), 30.2 (CH_2), 19.5 (CH_2), 12.6 (CH_3); LRMS (CI) 234 (100, $[M+H]^+$), 232 (100, $[M^{79}Br+H]^+$); HRMS (CI) calcd for $C_8H_{11}BrNO_2$ $[M^{79}Br+H]^+$ 231.9973, observed 231.9979.

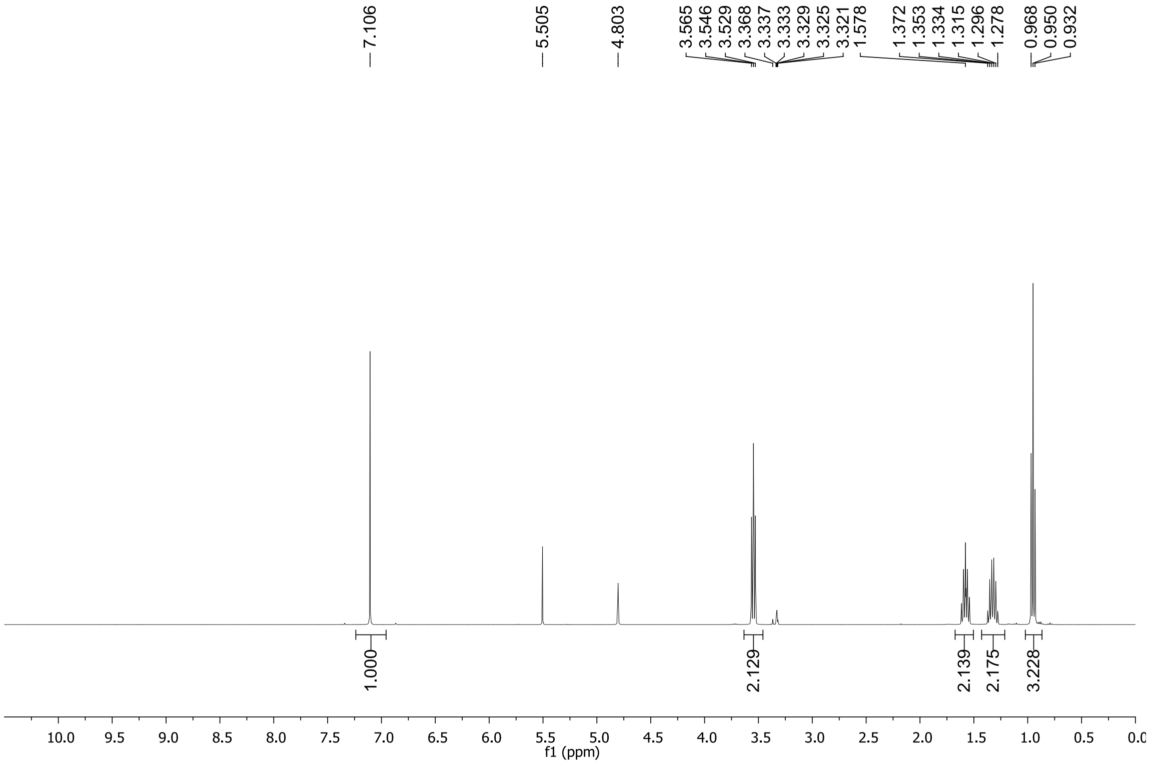

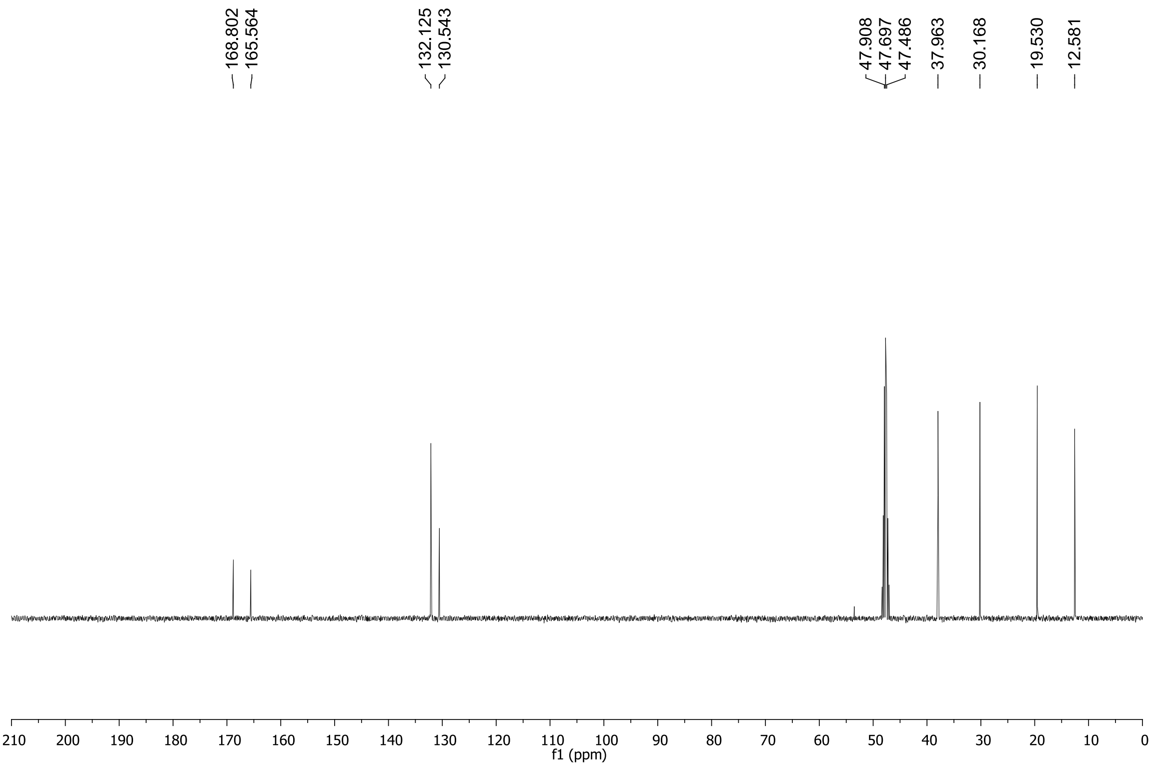

3-Bromo-1-methoxy-pyrrole-2,5-dione 2

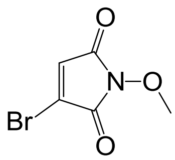

Purification by column chromatography (20% EtOAc/Petrol to neat EtOAc to 1% MeOH/CH_2Cl_2) gave 3-bromo-1-methoxy-pyrrole-2,5-dione **2** as a yellow oil (119 mg, 0.58 mmol, 58%): ^{1}H NMR ($CDCl_3$, 400 MHz) δ 6.85 (s, 1H), 3.97 (s, 3H); ^{13}C NMR ($CDCl_3$, 400 MHz) δ 163.3 (C), 160.6 (C), 129.9 (C), 128.9 (CH), 66.1 (CH_3); LRMS (CI) 206 (100, [$M^{81}Br+H]^+$), 204 (100, [$M^{79}Br+H]^+$); HRMS (CI) calcd for $C_5H_5O_3NBr$ [$M^{81}Br+H]^+$ 205.9453, observed 205.9447.

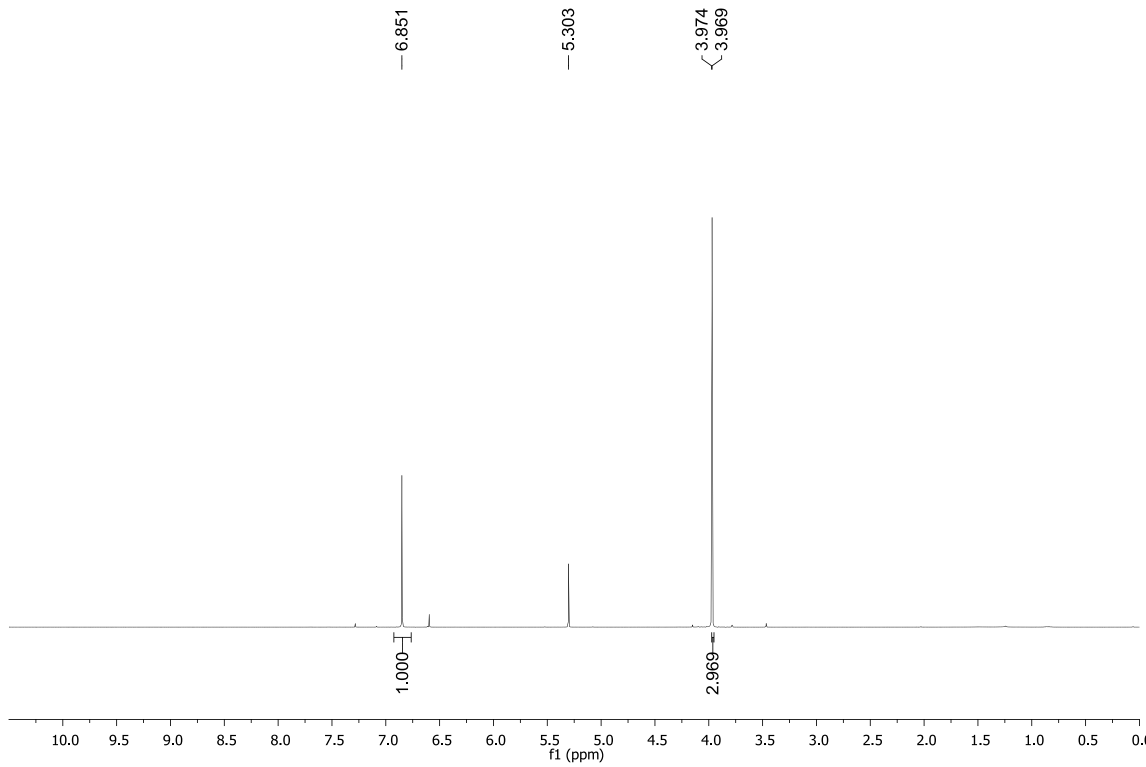
6.851
5.303
3.974
3.969
1.000
2.969
10.0 9.5 9.0 8.5 8.0 7.5 7.0 6.5 6.0 5.5 5.0 4.5 4.0 3.5 3.0 2.5 2.0 1.5 1.0 0.5 0.0
f1 (ppm)

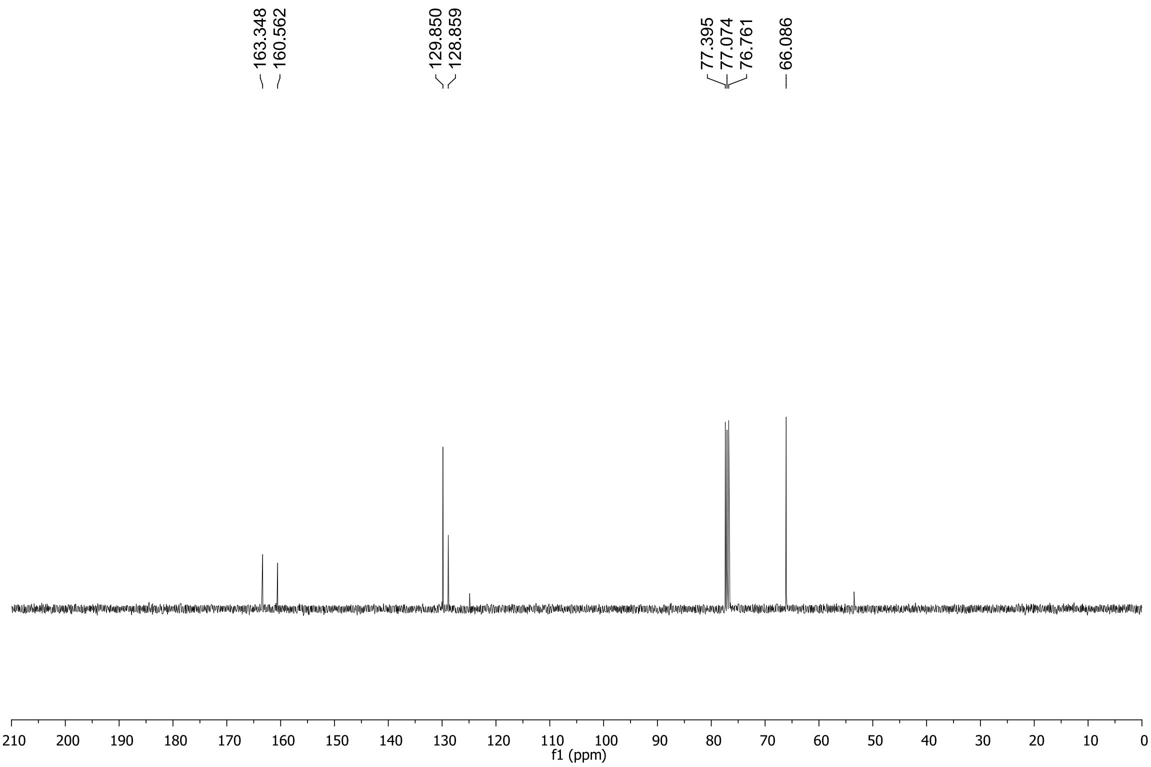
163.348
160.562
129.850
128.859
77.395
77.074
76.761
66.086
210 200 190 180 170 160 150 140 130 120 110 100 90 80 70 60 50 40 30 20 10 0
f1 (ppm)

3-Bromo-1-(2-methoxy-ethyl)-pyrrole-2,5-dione 3

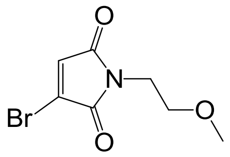

Purification by column chromatography (5% EtOAc/Petrol to neat EtOAc) gave 3-bromo-1-(2-methoxy-ethyl)-pyrrole-2,5-dione **3** as a yellow oil (126 mg, 0.54 mmol, 54%): ^{1}H NMR (400 MHz, CD_3OD) δ 7.14 (s, 1H), 3.74 (t, J = 5.5 Hz, 2H), 3.56 (t, J = 5.5 Hz, 2H), 3.32 (s, 3H); ^{13}C NMR (150 MHz, CD_3OD) δ 170.0 (C), 166.9 (C), 133.6 (CH), 132.0 (C), 70.1 (CH_2), 58.7 (CH_3), 38.9 (CH_2); LRMS (CI) 236 (85, $[M^{81}Br+H]^+$), 234 (85, $[M^{79}Br+H]^+$), 204 (100, $[(M^{81}Br-OCH_3)+H]^+$), 202 (100, $[(M^{79}Br-OCH_3)+H]^+$); HRMS (CI) calcd for $C_7H_9BrNO_3$ $[M^{79}Br+H]^+$ 233.9766, observed 233.9772.

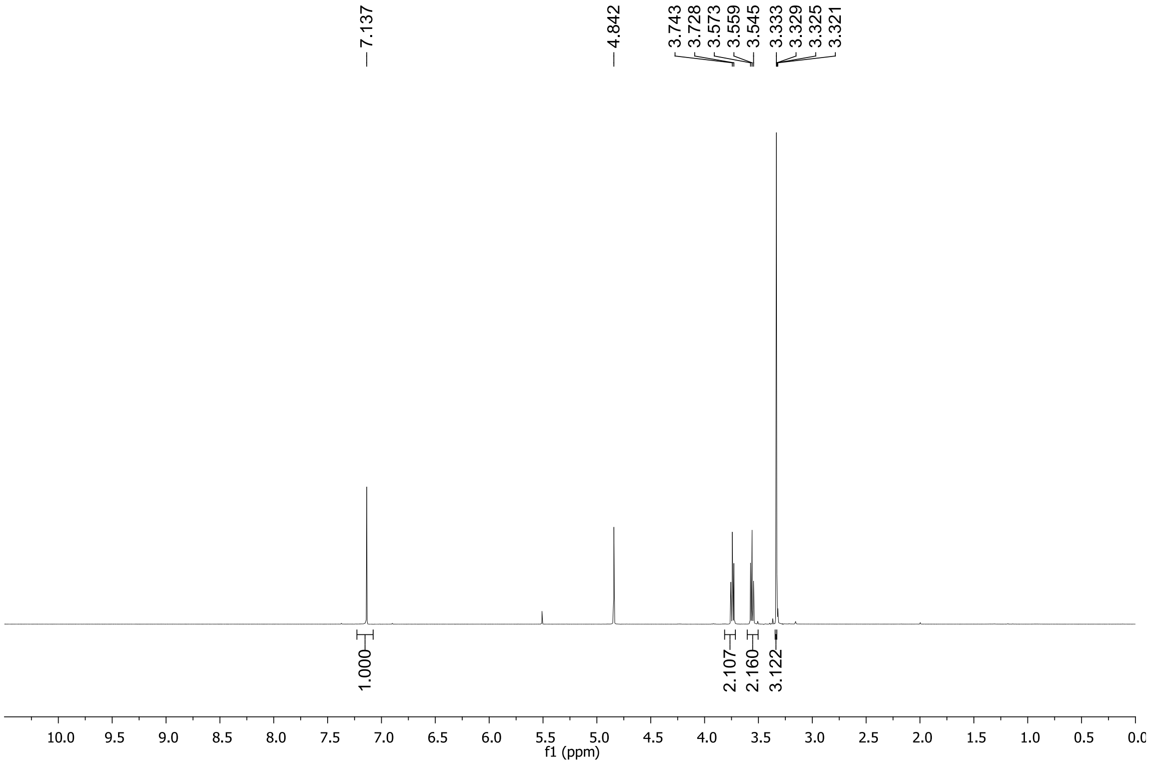

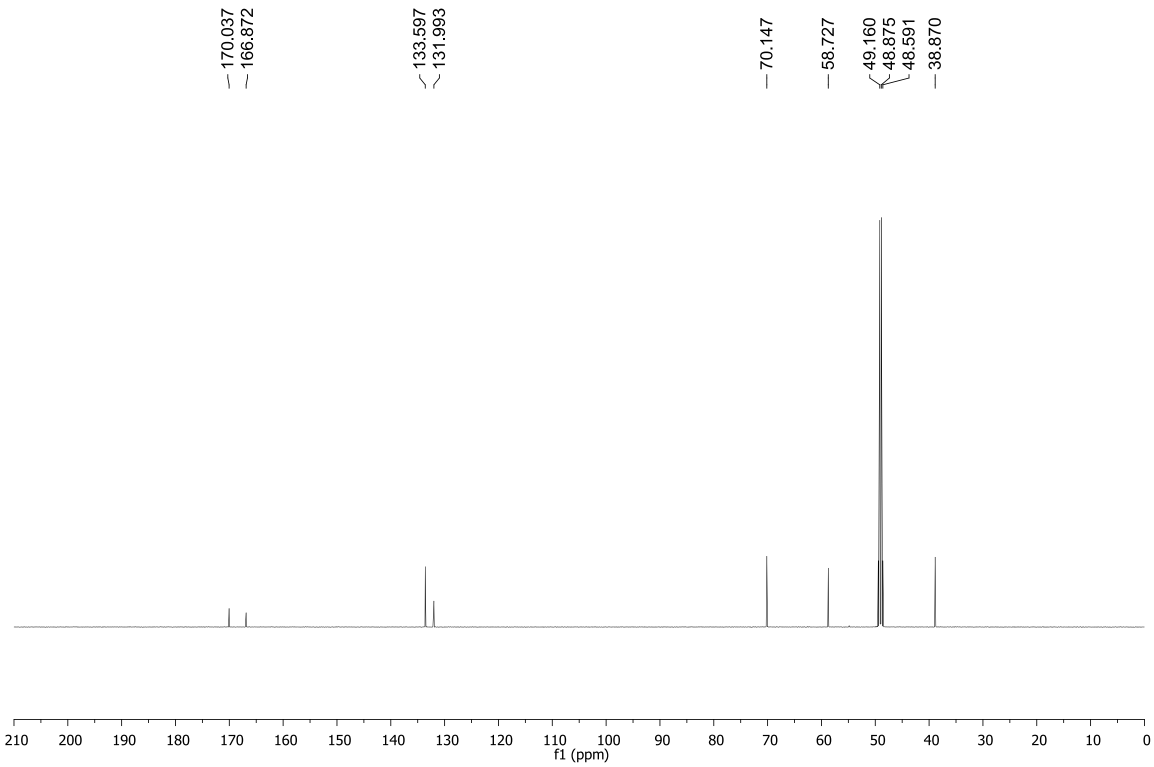

3-Bromo-1-(cyclohexylmethyl)-pyrrole-2,5-dione 4

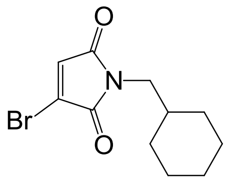

Purification by column chromatography (5% to 20% EtOAc/Petrol) gave 3-bromo-1-(cyclohexylmethyl)-pyrrole-2,5-dione **4** as a yellow solid (163 mg, 0.60 mmol, 60%): ^{1}H NMR (600 MHz, $CDCl_3$) δ 6.85 (s, 1H), 3.39 (d, J = 7.0 Hz, 2H), 1.72-1.60 (m, 6H), 1.22-1.10 (m, 3H), 0.98-0.88 (m, 2H); ^{13}C NMR (150 MHz, $CDCl_3$) δ 169.0 (C), 165.7 (C) 131.8 (CH), 131.3 (C), 45.1 (CH_2), 37.0 (CH), 30.7 (CH_2), 26.3 (CH_2), 25.7 (CH_2); LRMS (EI) 273 (65, $[M^{81}Br]^{+\cdot}$), 271 (65, $[M^{79}Br]^{+\cdot}$), 190 (100, $[(M^{81}Br\text{-}C_6H_{11})]^{+}$), 188 (100, $[(M^{79}Br\text{-} C_6H_{11})]^{+}$); HRMS (EI) calcd for $C_{11}H_{14}BrNO_2$ $[M^{79}Br]^{+\cdot}$ 271.0202, observed 271.0206.

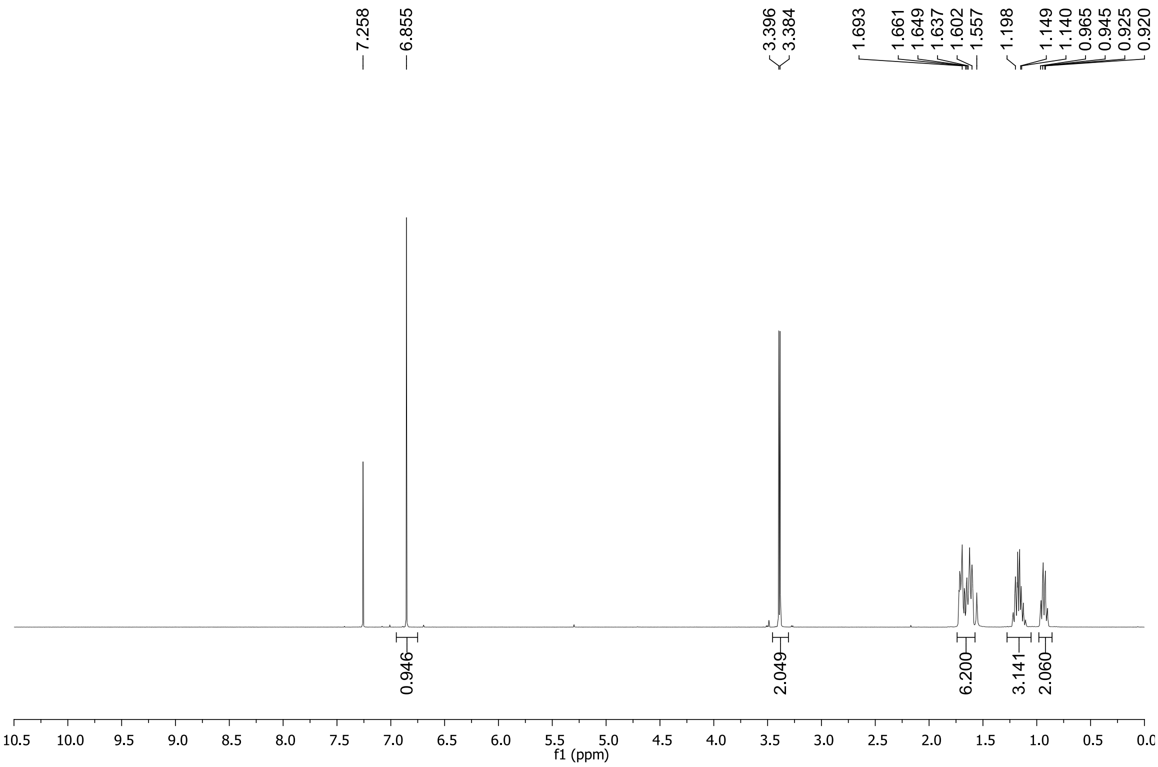
7.258
6.855
3.396
3.384
1.693
1.661
1.649
1.637
1.602
1.557
1.198
1.149
1.140
0.965
0.945
0.925
0.920
0.946
2.049
6.200
3.141
2.060
f1 (ppm)

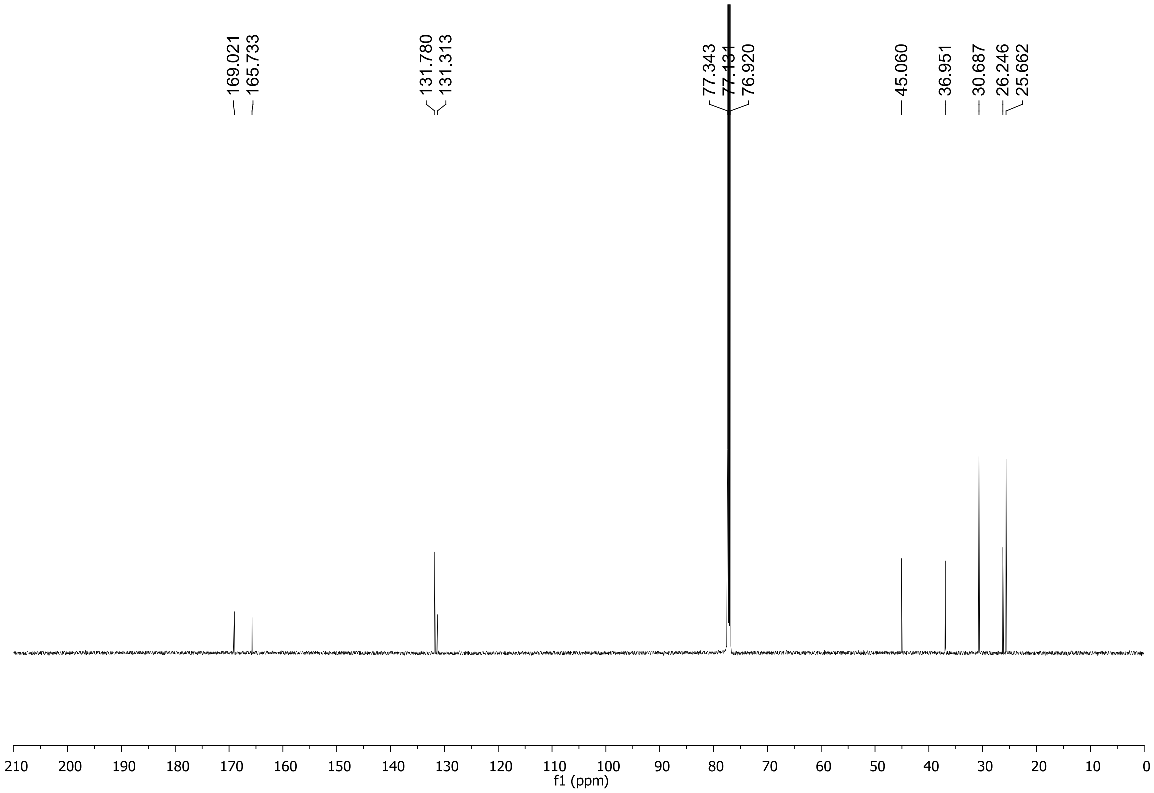
169.021
165.733
131.780
131.313
77.343
77.131
76.920
45.060
36.951
30.687
26.246
25.662
f1 (ppm)

Electronic Supplementary Material (ESI) for Organic & Biomolecular Chemistry
This journal is © The Royal Society of Chemistry 2013

Synthesis of biotin-decane-maleimide reagent 6

***tert*-Butyl (10-(5-((4*S*)-2-oxohexahydro-1H-thieno[3,4-d]imidazol-4-yl)pentanamido) decyl)carbamate**

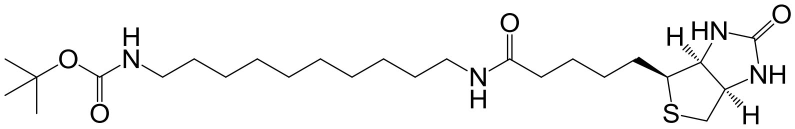

A solution of biotin (0.59 g, 2.42 mmol), HBTU (0.79 g, 2.10 mmol) and DIPEA (0.45 mL, 2.60 mmol) in DMF (15 mL) was stirred for 20 min at 21 °C before being added dropwise to a solution of *tert*-butyl (4-aminodecyl)carbamate [1] (400 mg, 1.61 mmol) in DMF (10 mL). The reaction mixture was stirred for 2 h at 21 °C. After this time, the DMF was removed *in vacuo* and the crude residue purified by column chromatography (2% to 10% MeOH/CH_2Cl_2) to yield tert-butyl (10-(5-((4*S*)-2-oxohexahydro-1H-thieno[3,4-d]imidazol-4-yl)pentanamido)decyl)carbamate as a white solid (641 mg, 1.29 mmol, 80%): ^{1}H NMR (600 MHz, CD_3OD) δ 4.49 (dd, J = 5.0, 7.0 Hz, 1H), 4.30 (dd, J = 5.0, 7.0 Hz, 1H), 3.22-3.19 (m, 1H), 3.16 (dt, J = 2.5, 7.0 Hz, 2H), 3.01 (q, J = 7.0 Hz, 2H), 2.93 (dd, J = 5.0, 12.5 Hz, 1H), 2.70 (d, J = 12.5 Hz, 1H), 2.19 (t, J = 7.0 Hz, 2H), 1.76-1.57 (m, 4H), 1.51-1.41 (m, 15H), 1.35-1.28 (m, 12H); ^{13}C NMR (150 MHz, CD_3OD) δ 176.0 (C), 166.1 (C), 158.6 (C), 79.8 (C), 63.4 (CH), 61.6 (CH), 57.0 (CH), 41.5 (CH_2), 41.0 (CH_2), 40.4 (CH_2), 36.8 (CH_2), 31.0 (CH_2), 30.7 (CH_2), 30.6 (CH_2), 30.4 (CH_2), 29.8 (CH_2), 29.5 (CH_2), 28.8 (CH_3), 28.0 (CH_2), 27.9 (CH_2), 27.0 (CH_2); IR (neat) 3305, 2937, 1692 cm^{-1}; LRMS (ES$^+$) 521 (100, [M+Na]$^+$); HRMS (ES$^+$) calcd for $C_{25}H_{46}N_4O_4NaS$ [M+Na]$^+$ 521.3137, observed 521.3126.

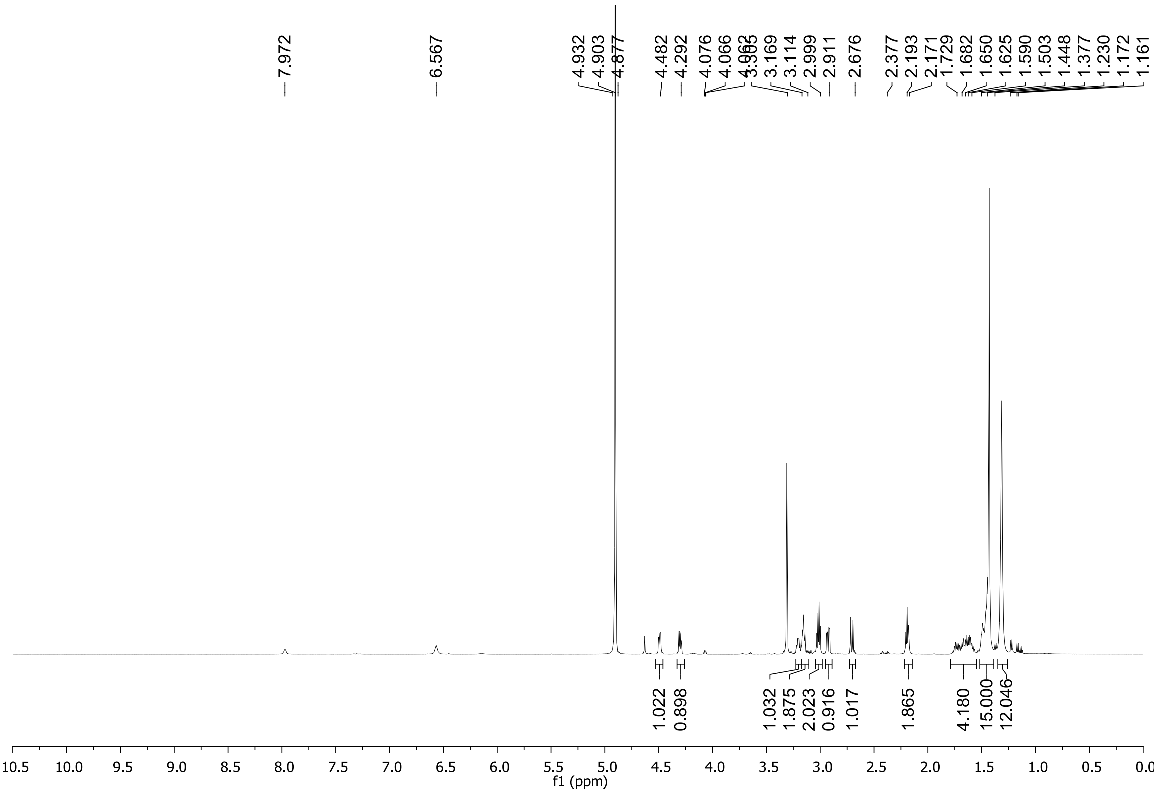

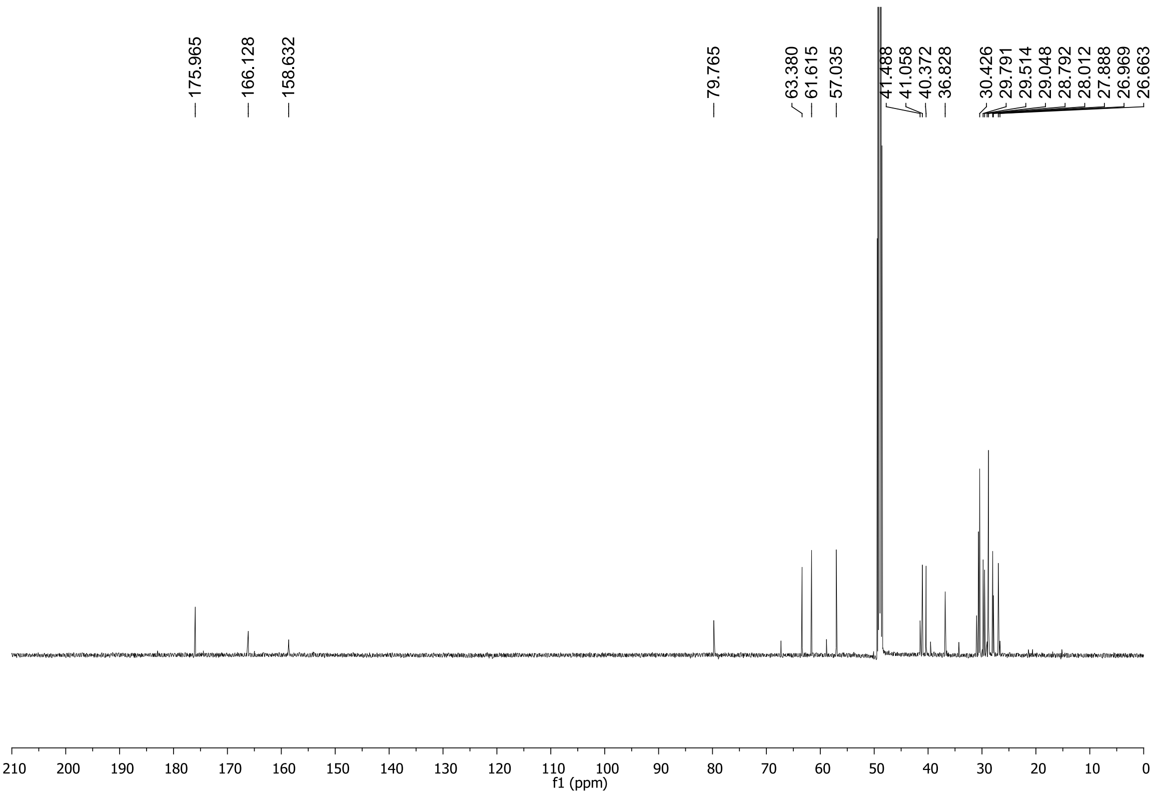

***N*-(10-(3-Bromo-2,5-dioxo-2,5-dihydro-1H-pyrrol-1-yl)decyl)-5-((4S)-2-oxohexahydro-1H-thieno[3,4-d]imidazol-4-yl)pentanamide 6**

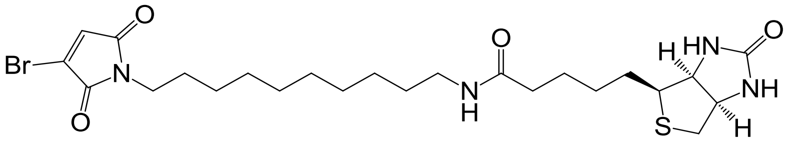

TFA (5 mL) was added to tert-butyl (10-(5-((4*S*)-2-oxohexahydro-1H-thieno[3,4-d]imidazol-4-yl)pentanamido)decyl)carbamate (125 mg, 0.25 mmol) and the reaction mixture stirred at 21 °C for 15 h. After this time, toluene was added (5 mL) and the solvent removed *in vacuo* to give crude 10-(5-((4S)-2-oxohexahydro-1H-thieno[3,4-d]imidazol-4-yl)pentanamido)decan-1-amonium 2,2,2-trifluoroacetate. 3-Bromo-maleic anhydride (45.0 mg, 0.25 mmol) was added in one portion to a solution of crude 10-(5-((4S)-2-oxohexahydro-1H-thieno[3,4-d]imidazol-4-yl)pentanamido)decan-1-amonium 2,2,2-trifluoroacetate in AcOH (10 mL) and the reaction was heated at reflux for 3 h. After cooling the reaction mixture to room temperature toluene was added (20 mL), the solvents were removed *in vacuo* (x2) and the crude residue was purified by column chromatography (2% to 10% MeOH/CH_2Cl_2) to afford *N*-(10-(3-bromo-2,5-dioxo-2,5-dihydro-1H-pyrrol-1-yl)decyl)-5-((4*S*)-2-oxohexahydro-1H-thieno[3,4-d]imidazol-4-yl)pentanamide **6** as a white solid (70 mg, 0.13 mmol, 68% yield): ^{1}H NMR ($CDCl_3$, 600 MHz) δ 6.86 (s, 1H), 6.37-6.34 (br m, 1H), 6.04-5.98 (br m, 1H), 5.74-5.68 (br m, 1H), 4.51-4.49 (m, 1H), 4.31-4.29 (m, 1H), 3.53 (t, J = 7.0 Hz, 2H), 3.21-3.12 (m, 3H), 2.89 (dd, J = 4.5, 13.0 Hz, 1H), 2.73 (d, J = 13.0 Hz, 1H), 2.18 (t, J = 7.5 Hz, 2H), 1.74-1.54 (m, 6H), 1.47-1.39 (m, 4H), 1.28-1.22 (m, 12H); ^{13}C NMR ($CDCl_3$, 150 MHz) δ 173.2 (C), 168.8 (C), 165.5 (C), 164.3 (C), 131.9 (CH), 131.4 (C), 61.9 (CH), 60.2 (CH), 55.7 (CH), 40.6 (CH_2), 39.5 (CH_2), 39.0 (CH_2), 36.1 (CH_2), 29.7 (CH_2), 29.5 (CH_2), 29.4 (CH_2), 29.4 (CH_2), 29.1 (CH_2), 28.5 (CH_2), 28.4 (CH_2), 28.2 (CH_2), 27.0 (CH_2), 26.7 (CH_2), 25.8 (CH_2); IR (neat) 3352, 2975, 1732, 1584

cm^{-1}; LRMS (ES^{+}) 581 (100, [M^{79}Br+Na]$^{+}$), 579 (100, [M^{79}Br+Na]$^{+}$); HRMS (CI) calcd for $C_{24}H_{38}N_4O_4SBr$ [M^{79}Br+H]$^{+}$ 577.1797, observed 577.1769.

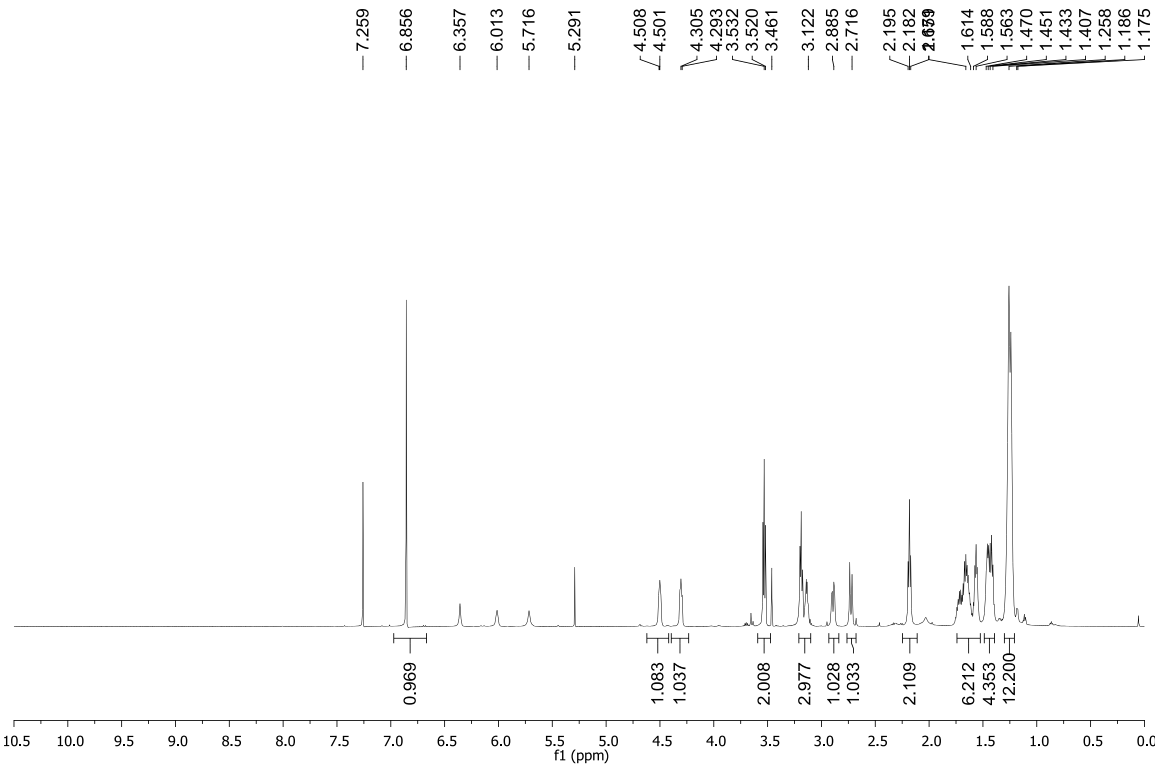

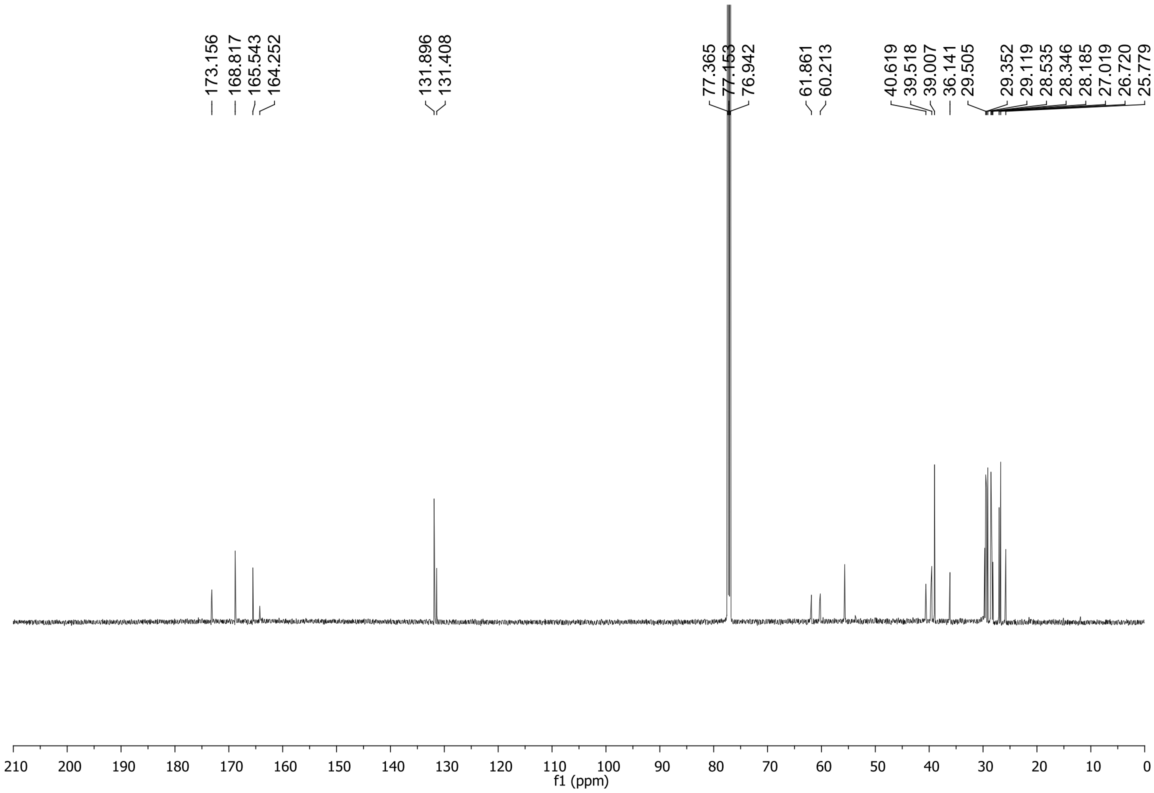

Small molecule studies

(*R*)-Methyl 2-((*tert*-butoxycarbonyl)amino)-3-((1-methyl-2,5-dioxo-2,5-dihydro-1H-pyrrol-3-yl)thio)propanoate 10

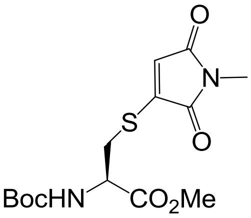

To a stirring solution of *N*-Boc-Cys-OMe (320 mg, 1.36 mmol) and NaOAc (820 mg, 4.08 mmol) in MeOH (10 mL) was added a solution of N-methyl bromomaleimide (258 mg, 1.36 mmol) in MeOH (10 mL) over a period of 10 min. After this time, the solvents were removed *in vacuo* and the crude residue was purified by column chromatography (10% to 30% EtOAc/Petrol) to afford (*R*)-methyl 2-((tert-butoxycarbonyl)amino)-3-((1-methyl-2,5-dioxo-2,5-dihydro-1H-pyrrol-3-yl)thio)propanoate **10** as a pale white powder (393 mg, 1.14 mmol, 84%): m.p. 101-103 °C; ^{1}H NMR ($CDCl_3$, 600 MHz) δ 6.29 (s, 1H), 5.41 (d, 1H, J = 6.5 Hz), 4.69-4.67 (m, 1H), 3.81 (s, 3H), 3.49 (dd, 1H, J = 13.5 and 5.0 Hz), 3.37 (dd, 1H, J = 13.5 and 5.0 Hz), 3.02 (s, 3H), 1.46 (s, 9H); ^{13}C NMR ($CDCl_3$, 125 MHz) δ 170.5 (C), 169.8 (C), 168.0 (C), 155.3 (C), 150.3 (C), 118.7 (CH), 81.2 (C), 53.5 (CH_3), 53.0 (CH), 34.1 (CH_2), 28.6 (CH_3), 24.4 (CH_3); IR (solid) 3368, 2977, 1695 cm^{-1}; LRMS (ES^+) 367 (46, $[M+Na]^+$), 344 (100, $[M]^+$); HRMS (ES^+) calcd for $C_{14}H_{20}N_2O_6NaS$ $[M+Na]^+$ 367.0940, observed 367.0931; $^{20}\alpha_D$ = -18.6° (c 1.0, MeOH).

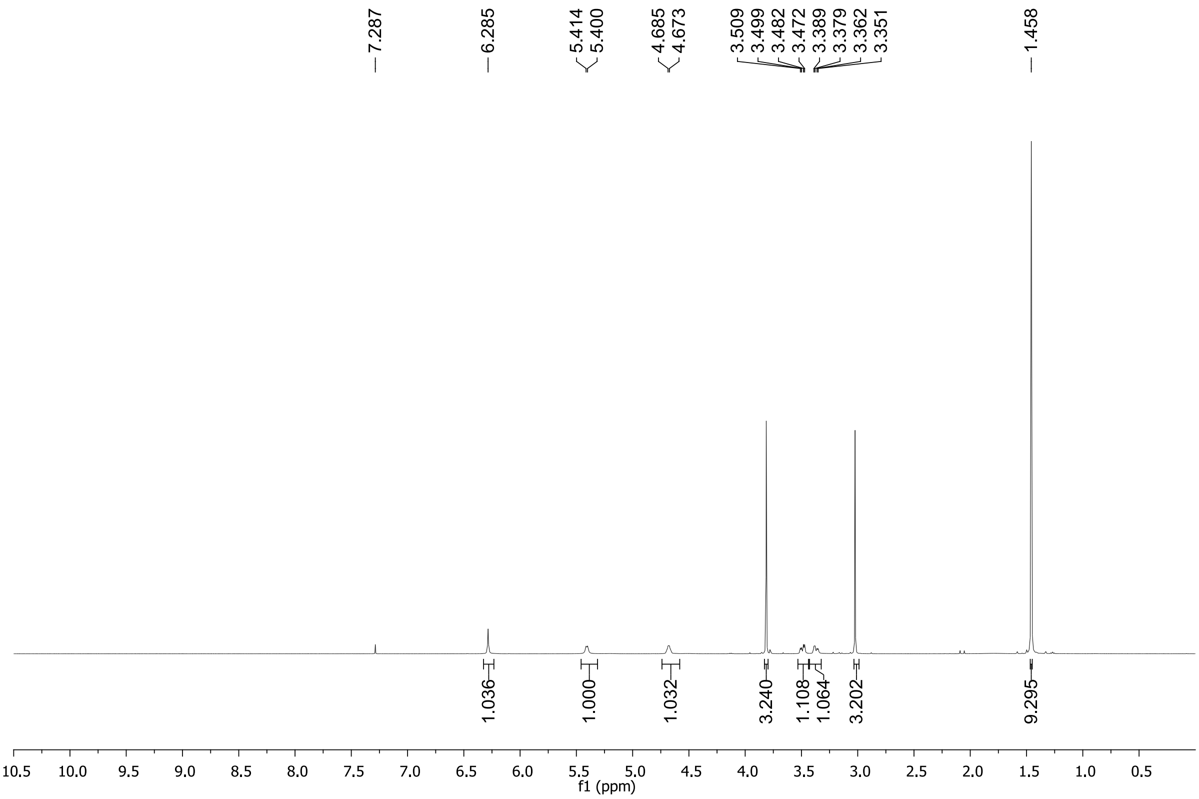

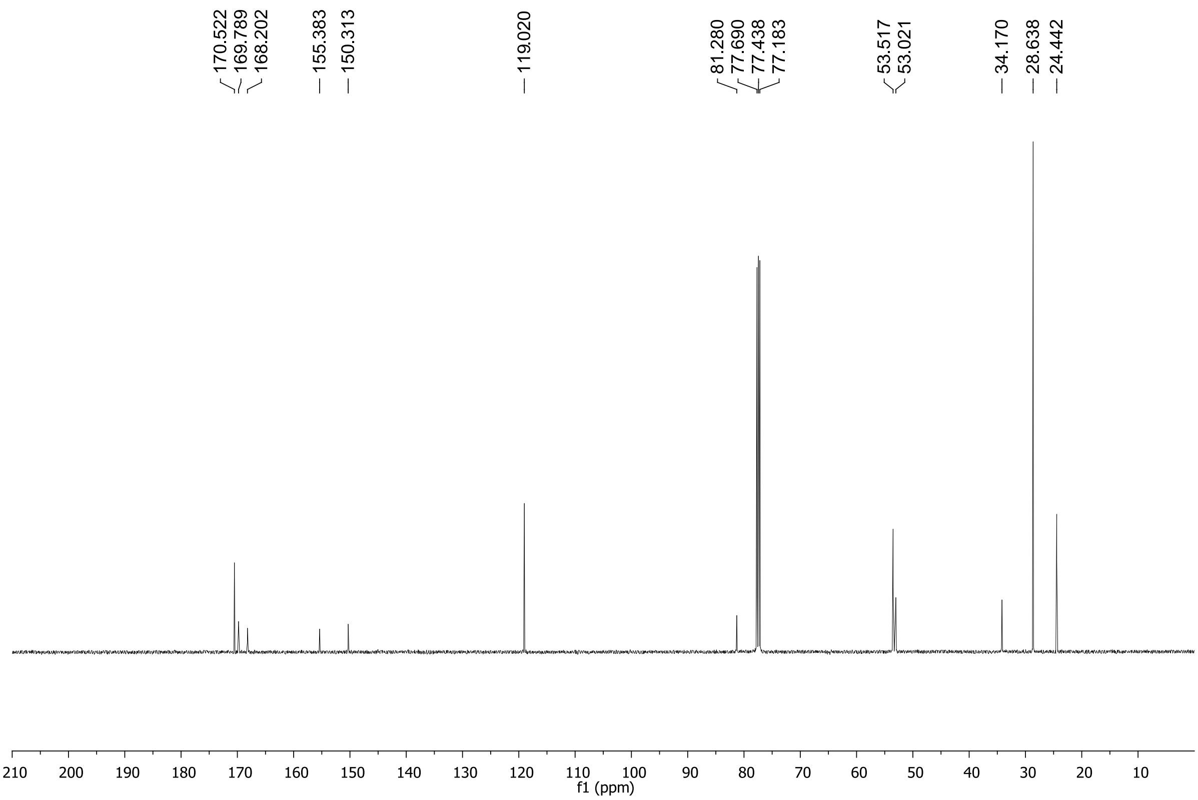

7-Methyl-1,4-dithia-7-azaspiro[4.4]nonane-6,8-dione 11

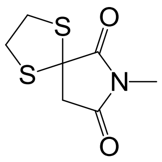

To a solution of (*R*)-Methyl 2-((tert-butoxycarbonyl)amino)-3-((1-methyl-2,5-dioxo-2,5-dihydro-1H-pyrrol-3-yl)thio)propanoate **10** (40 mg, 0.12 mmol) in a mixture of DMF (1.5 mL) and sodium phosphate buffer (100 mM, pH 8.0, 13 mL) was added 1,2-ethanedithiol (11 mg, 0.12 mmol) in DMF (1.5 mL). The reaction mixture was heated at 37 °C for 24 h. The aqueous reaction mixture was extracted with EtOAc (2 x 25 mL). The organics were combined and washed with saturated aqueous LiCl (3 x 25 mL), saturated aqueous NaCl (25 mL), dried ($MgSO_4$) and the solvent removed *in vacuo*. The crude residue purified by column chromatography (10% to 40% EtOAc/Petrol) to afford 7-methyl-1,4-dithia-7-azaspiro[4.4]nonane-6,8-dione **11** as a white solid (22 mg, 0.11 mmol, 95%): ^{1}H NMR ($CDCl_3$, 600 MHz) δ 3.77-3.71 (m, 2H), 3.59-3.52 (m, 2H), 3.26 (s, 2H), 3.02 (s, 3H); ^{13}C NMR ($CDCl_3$, 150 MHz) δ 178.1 (C), 173.1 (C), 60.0 (C), 42.2 (CH_2), 41.1 (CH_2), 25.7 (CH_3); IR (solid) 2935, 1736, 1434 cm^{-1}; LRMS (ES^+) 203 (100, $[M]^+$); HRMS (ES^+) calcd for $C_7H_9NO_2S_2$ $[M]^+$ 203.0069, observed 203.0067.

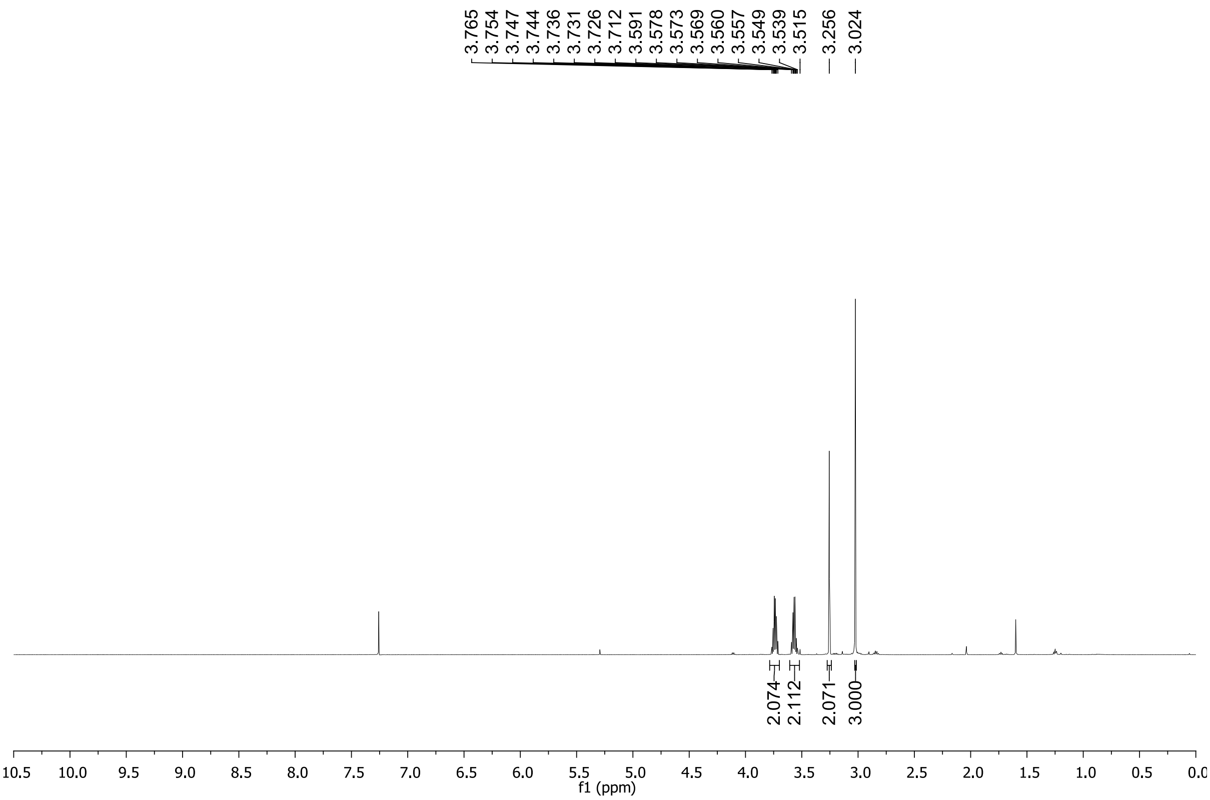

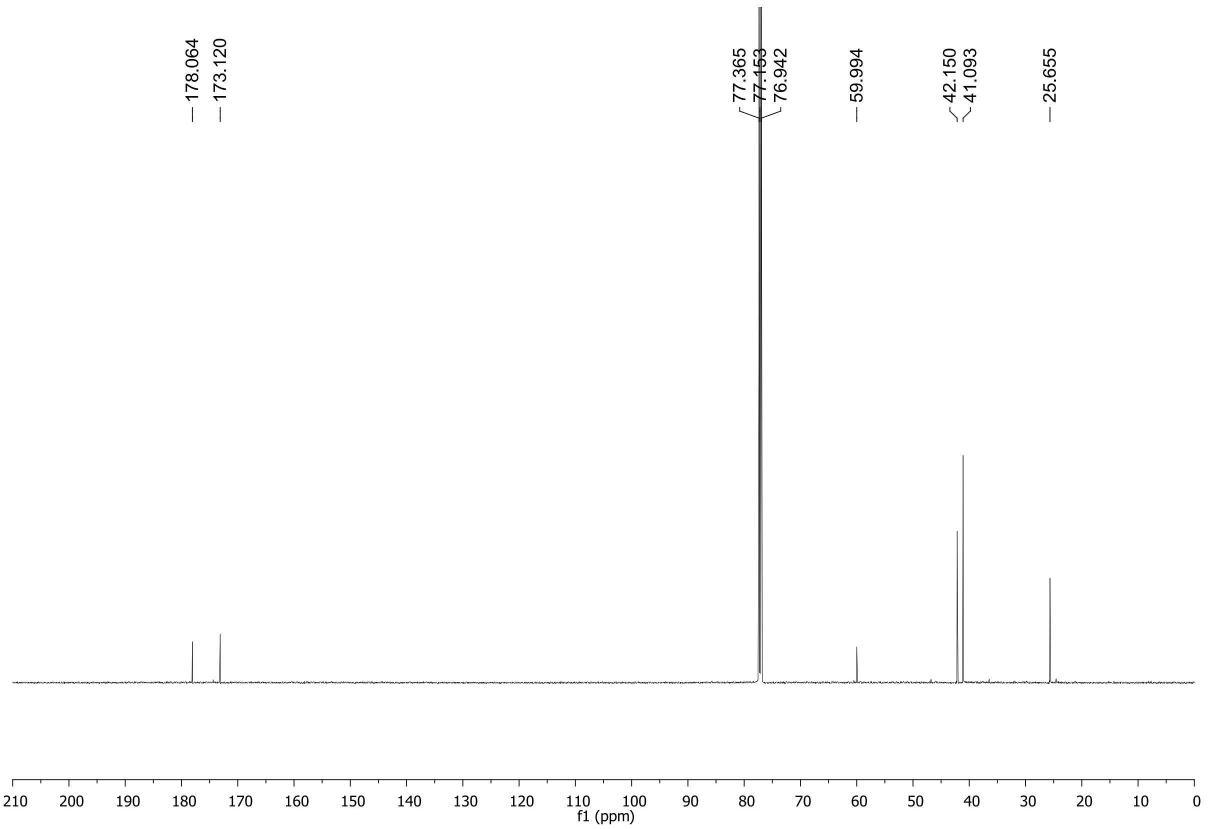

6-Methyldihydro-4a*H*-[1,4]dithiino[2,3-*c*]pyrrole-5,7(6*H*,7a*H*)-dione 12

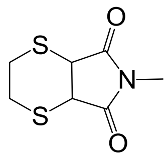

To a solution of *N*-methyl bromomaleimide (40 mg, 0.21 mmol) in a mixture of DMF (1.5 mL) and sodium phosphate buffer (100 mM, pH 8.0, 13 mL) was added 1,2-ethanedithiol (20 mg, 0.21 mmol) in DMF (1.5 mL). The reaction mixture was heated at 37 °C for 90 min. The aqueous reaction mixture was extracted with EtOAc (2 x 25 mL). The organics were combined and washed with saturated aqueous LiCl (3 x 25 mL), saturated aqueous NaCl (25 mL), dried ($MgSO_4$), the solvent removed *in vacuo* and the crude residue purified by column chromatography (10% to 30% EtOAc/Petrol) to afford 6-methyldihydro-4a*H*-[1,4]dithiino[2,3-*c*]pyrrole-5,7(6*H*,7a*H*)-dione **12** as a white solid (15 mg, 0.07 mmol, 35%): ^{1}H NMR ($CDCl_3$, 600 MHz) δ 4.05 (s, 2H), 3.10 (s, 3H), 2.88-2.79 (m, 4H); ^{13}C NMR ($CDCl_3$, 150 MHz) δ 174.3 (C), 40.8 (CH_3), 25.8 (CH), 24.8 (CH_2); IR (solid) 2985, 1737, 1447, 1373 cm^{-1}; LRMS (ES$^+$) 203 (100, [M]$^+$); HRMS (ES$^+$) calcd for $C_7H_9NO_2S_2$ [M]$^+$ 203.0069, observed 203.0070.

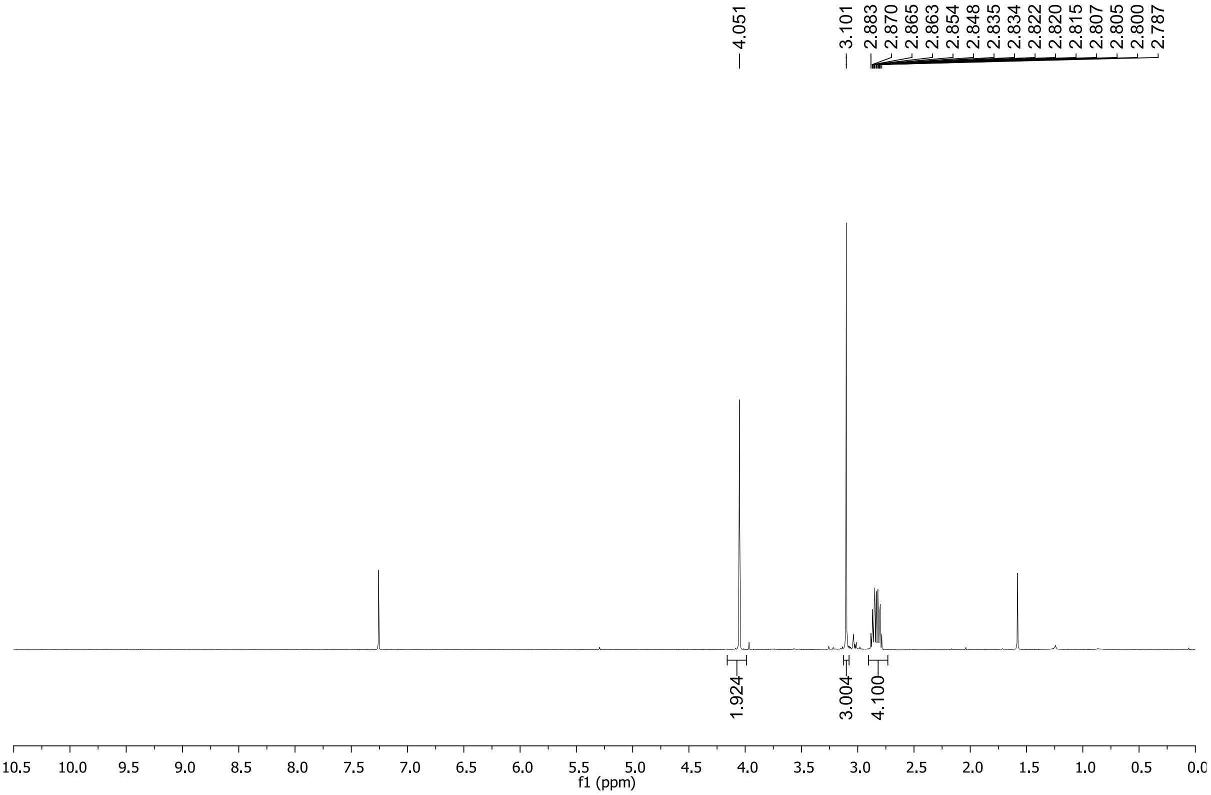

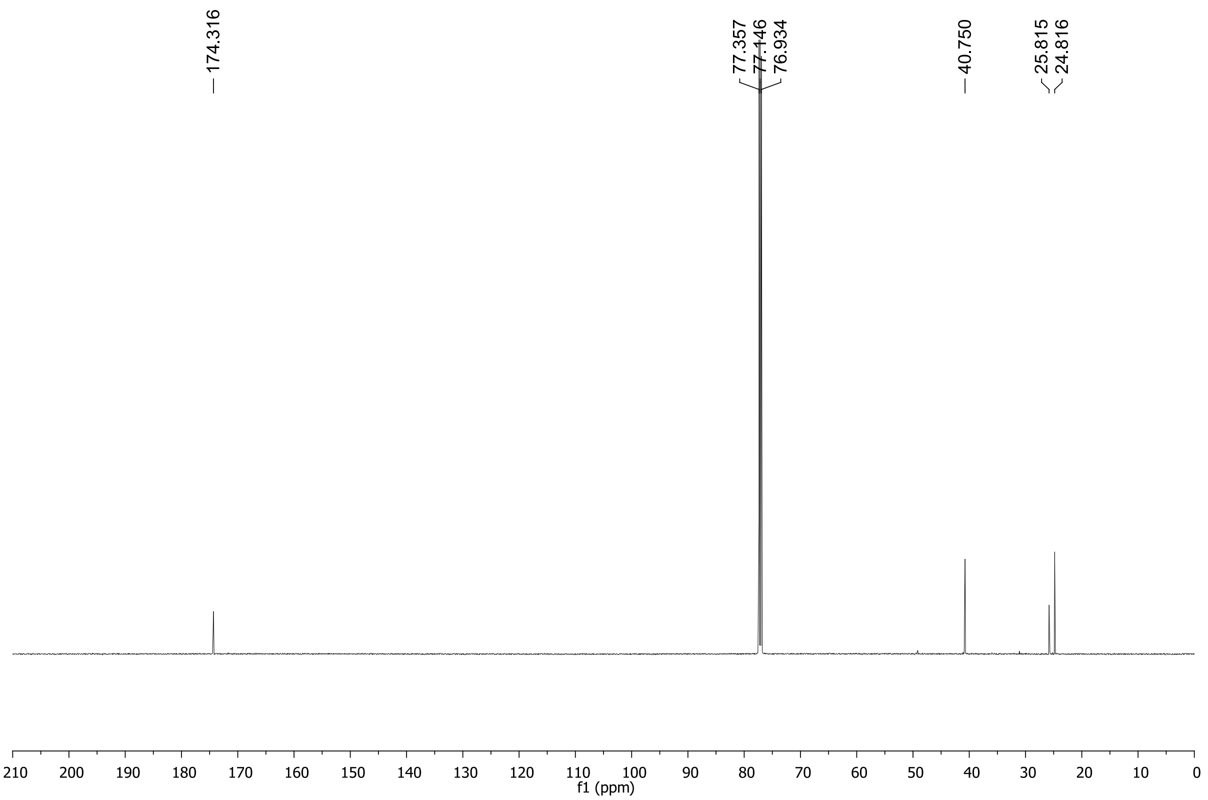

Cloning and expression of Grb2 SH2 (L111C) 5

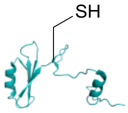

The cloning and expression of this protein was carried out by the method described by Caddick.[2] The mass of the monomeric protein **5** (mass 14164) was obtained using LC-MS:

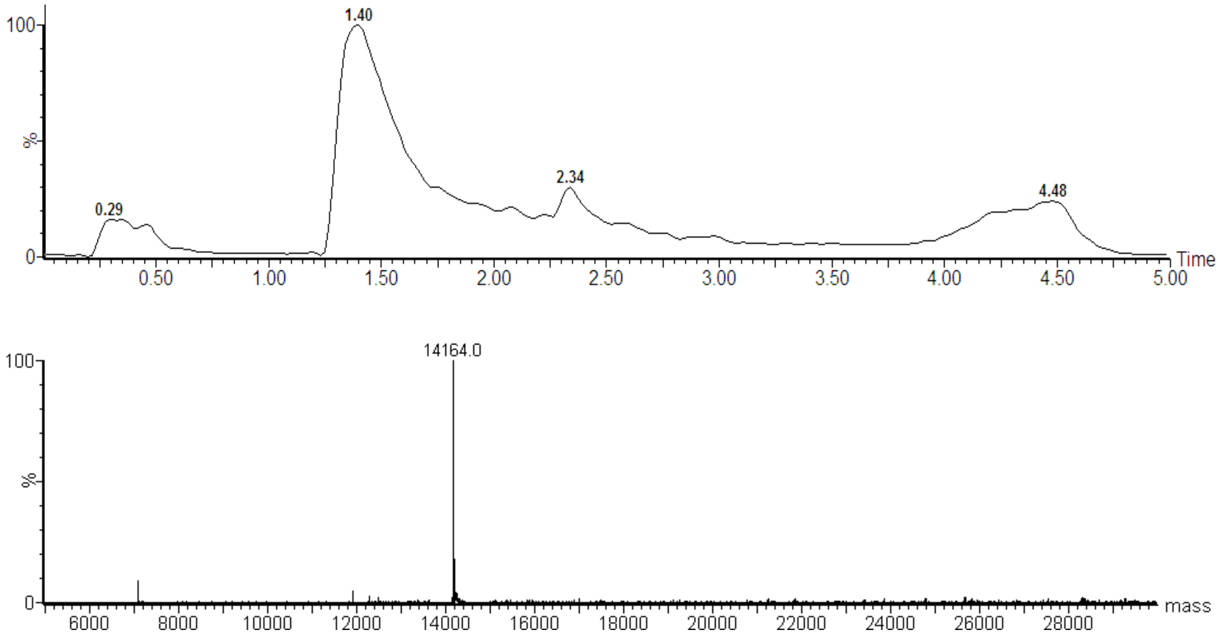

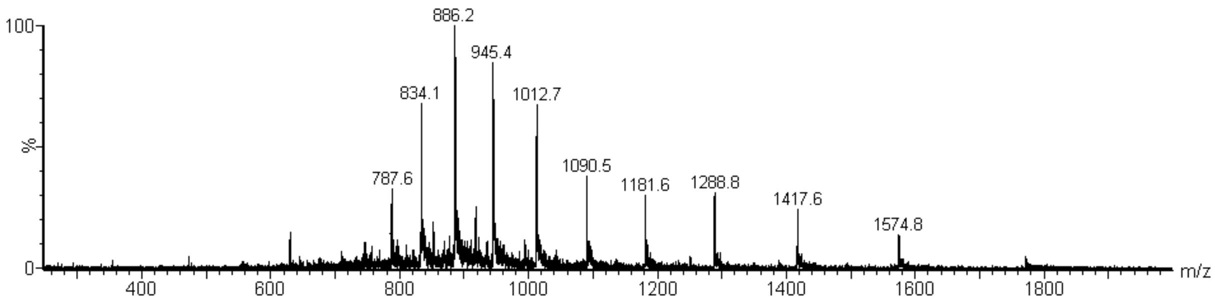

Cloning and expression of GFP (S147C) 7

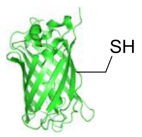

The cloning and expression of this protein was carried out by the method described by Caddick.[3] The mass of the monomeric protein **7** (mass 29332) was obtained using LC-MS:

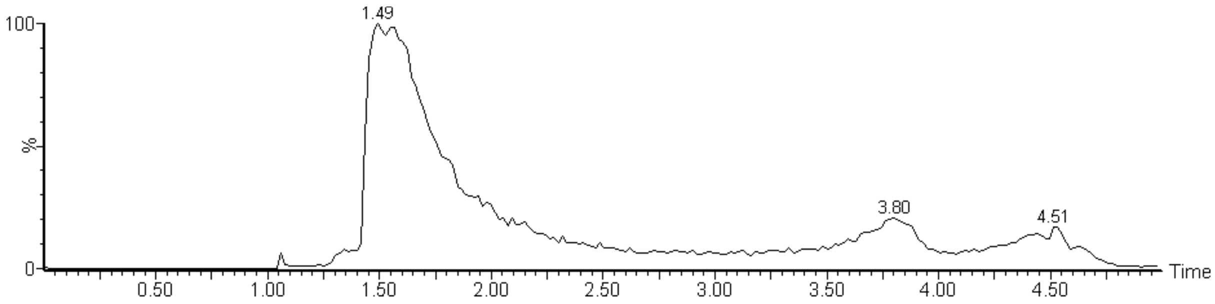

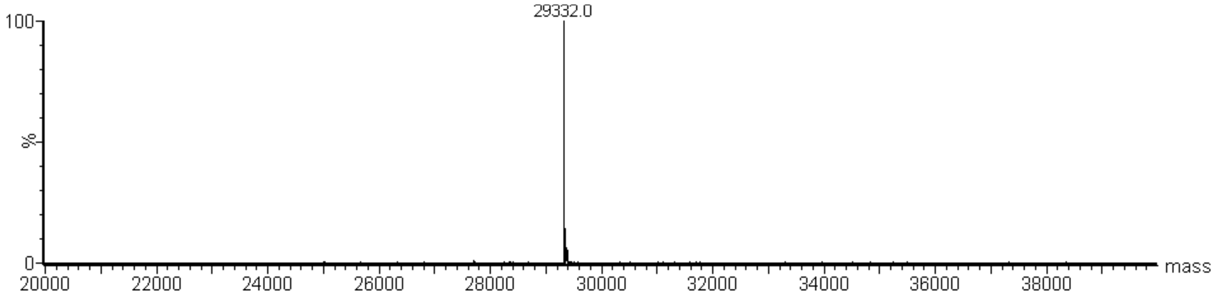

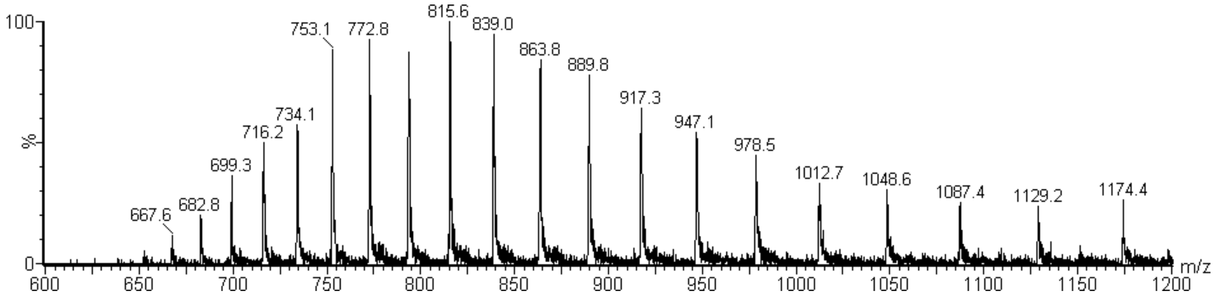

General Procedure for protein modification with bromomaleimide reagents

To a solution of protein (100 μL, [protein] 1.0 mg/mL, 100 mM sodium phosphate, 150 mM NaCl, pH 8.0) at 0 °C was added bromomaleimide (5 μL solution in DMF, 1 equivalent). The mixture was maintained at 0 °C for 1 h and analysed by LCMS.

Grb2 SH2 (L111C) 5 conjugate with bromomaleimide 1

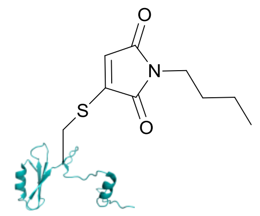

Expected Mass: 14316; Observed Mass: 14316

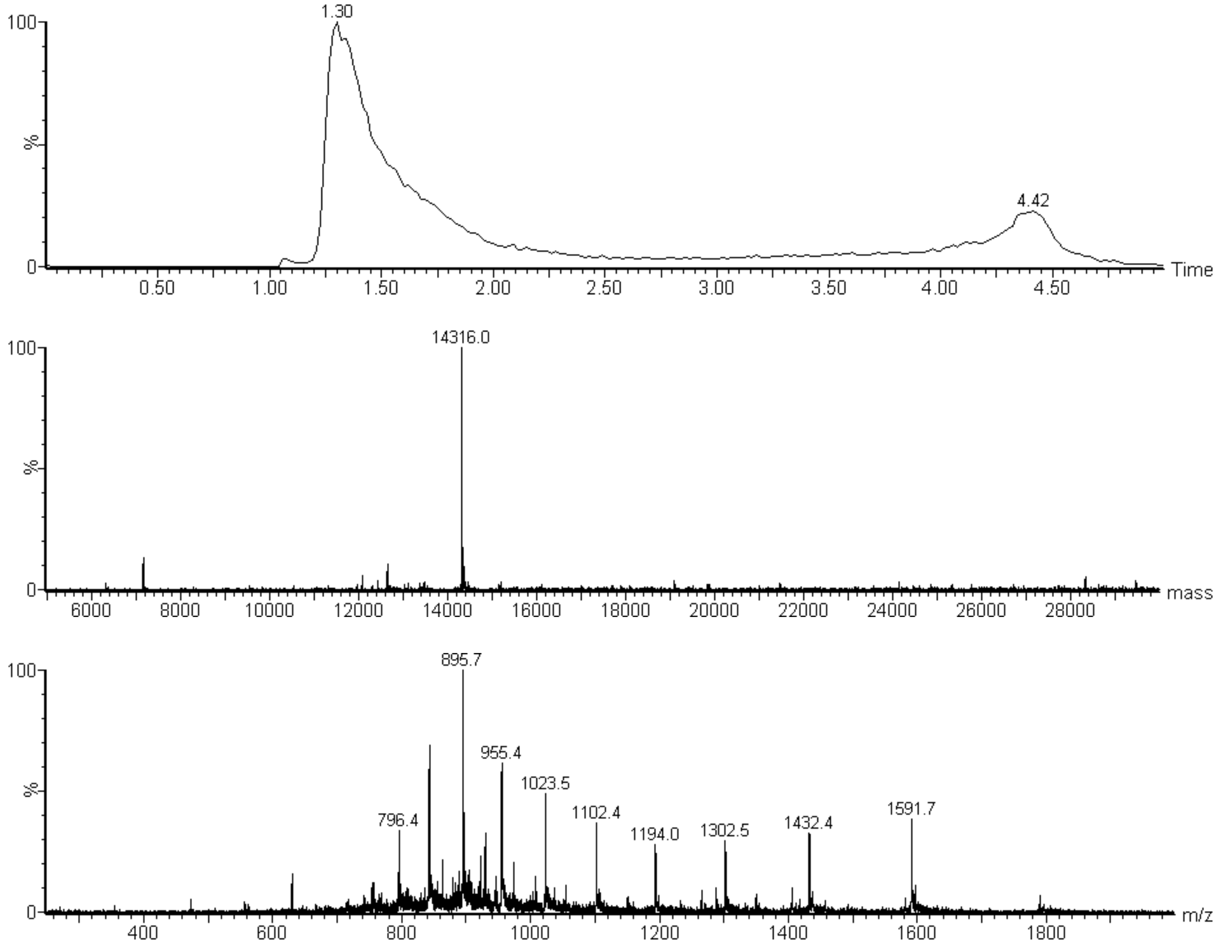

Grb2 SH2 (L111C) 5 conjugate with bromomaleimide 2

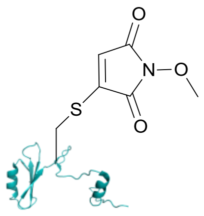

Expected Mass: 14290; Observed Mass: 14290

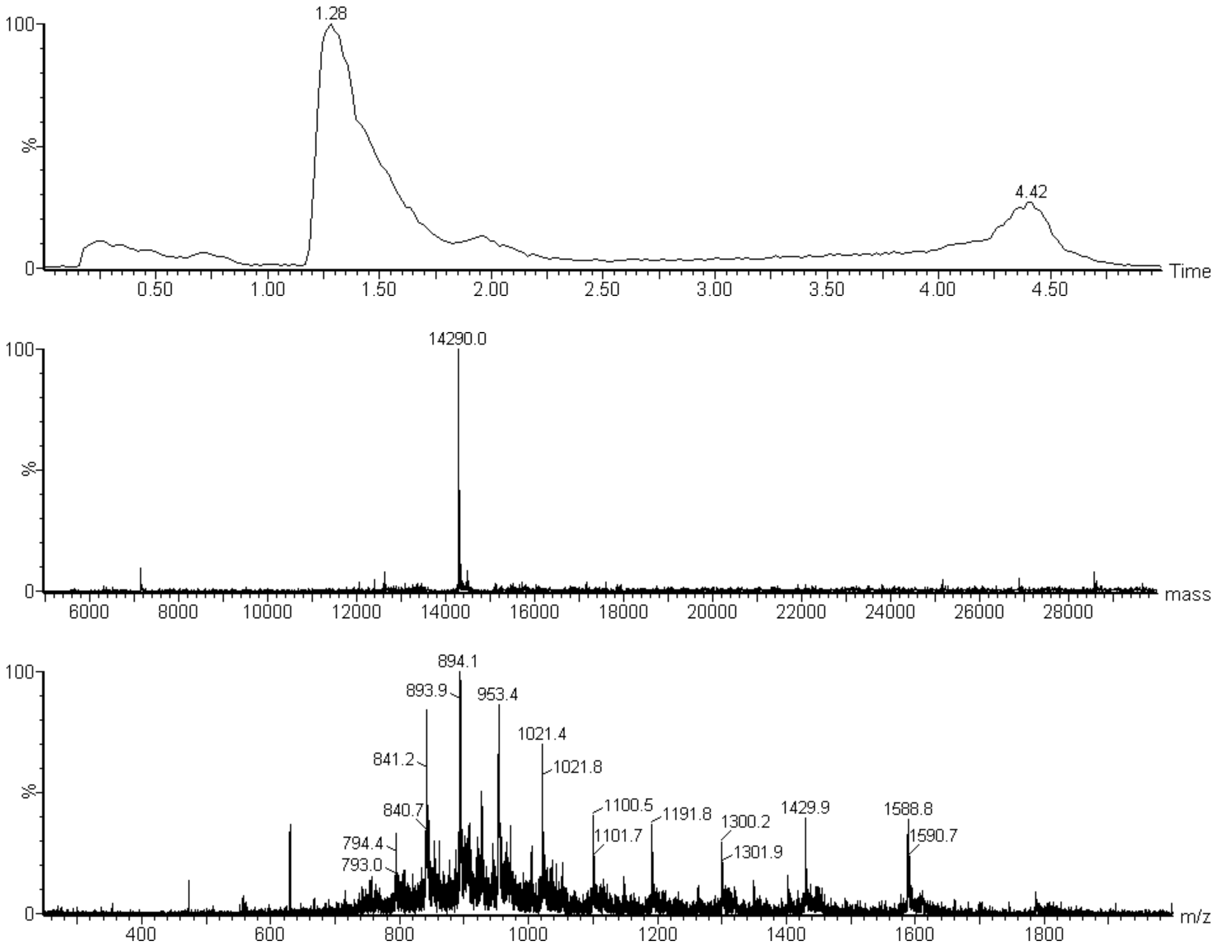

Grb2 SH2 (L111C) 5 conjugate with bromomaleimide 3

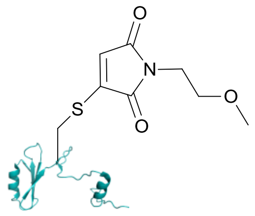

Expected Mass: 14318; Observed Mass: 14318

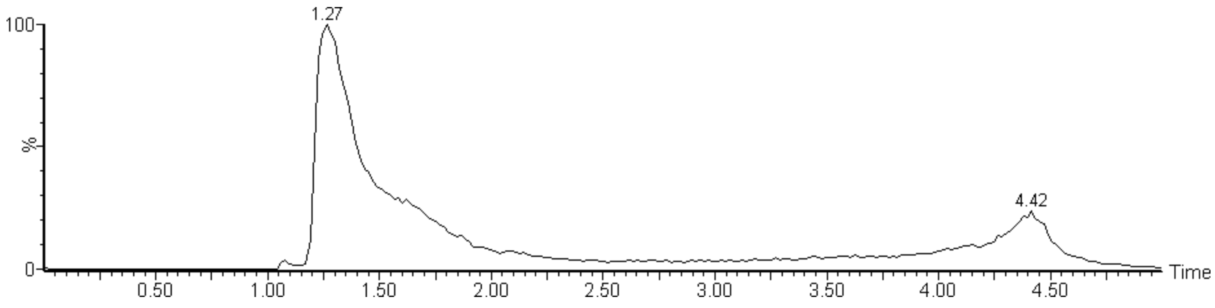

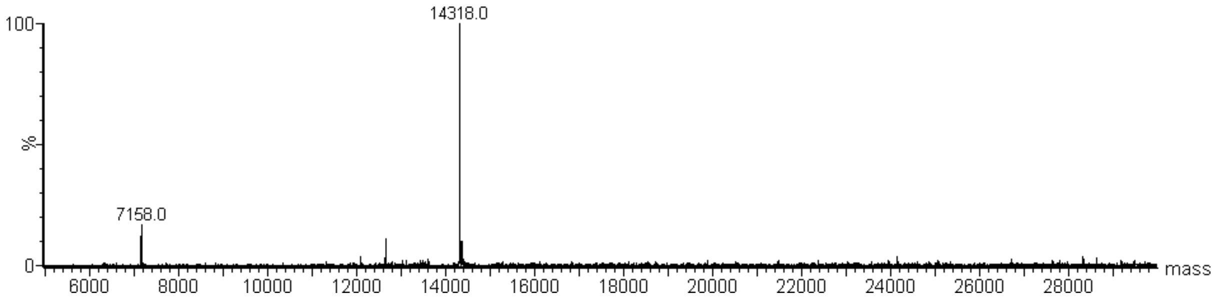

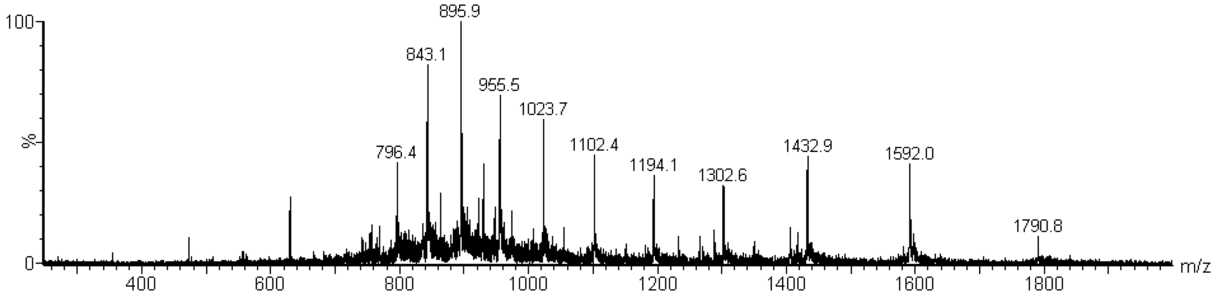

Grb2 SH2 (L111C) 5 conjugate with bromomaleimide 4

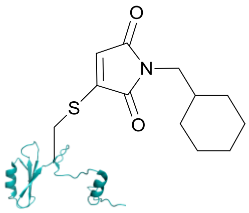

Expected Mass: 14356; Observed Mass: 14356

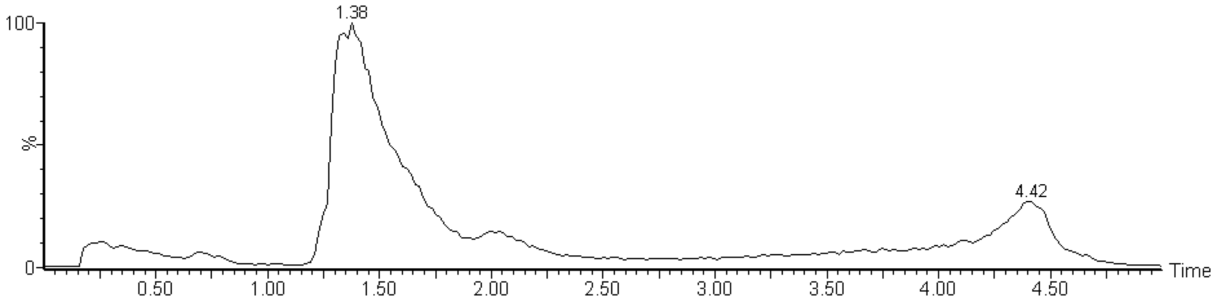

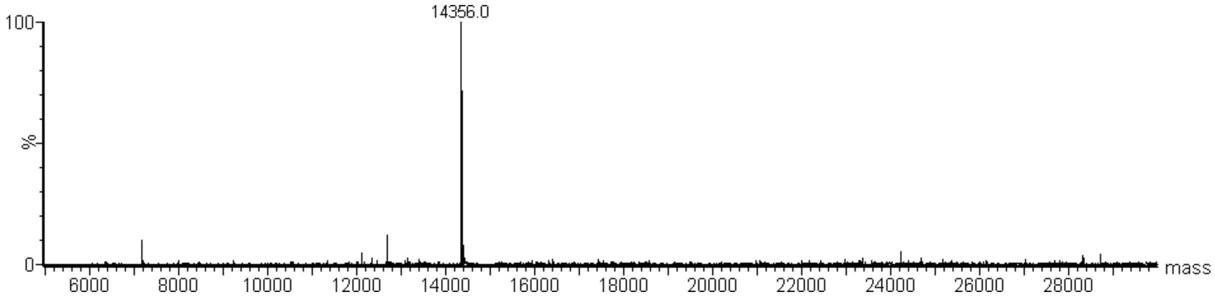

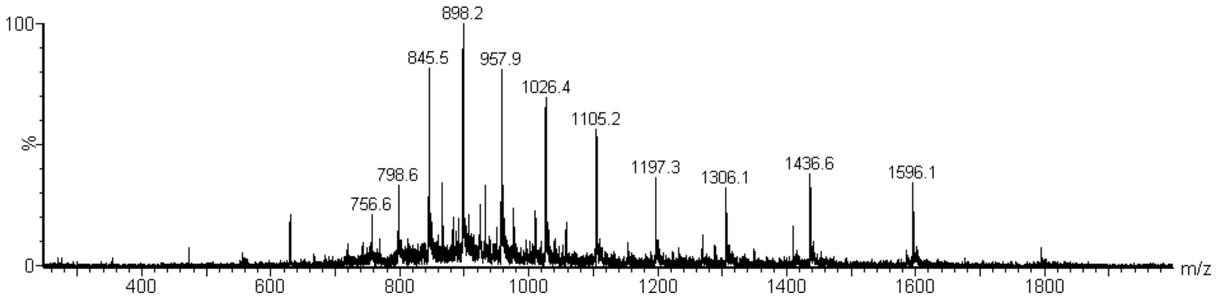

Grb2 SH2 (L111C) 5 conjugate with bromomaleimide 6

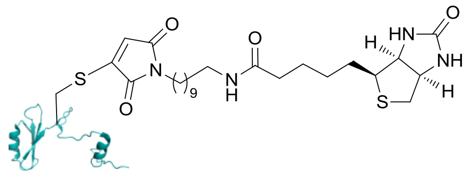

Expected Mass: 14641; Observed Mass: 14644

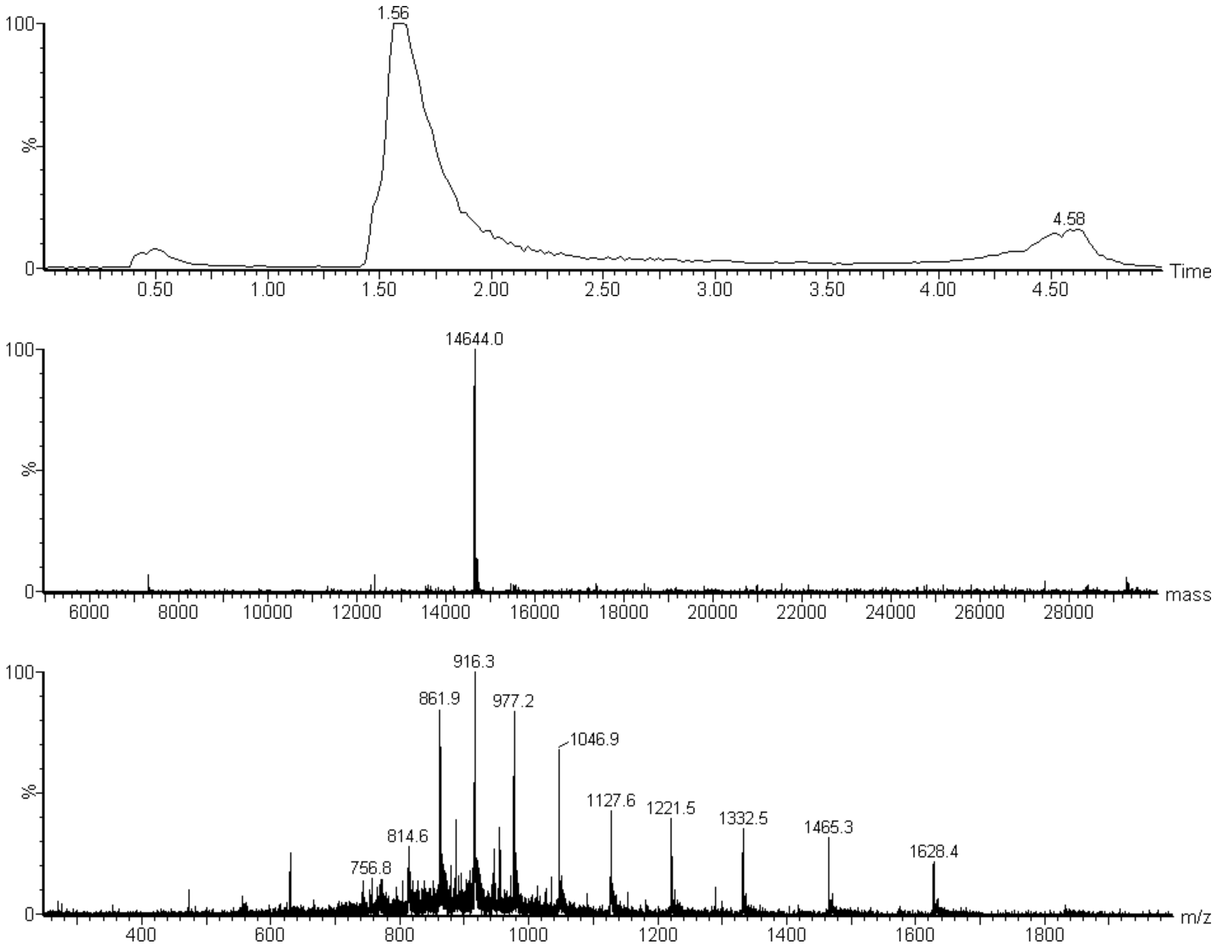

GFP (S147C) 7 conjugate with bromomaleimide 6

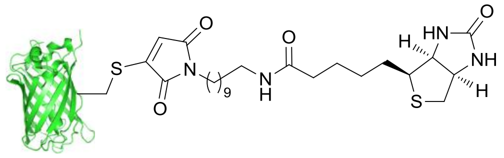

Expected Mass: 29809; Observed Mass: 29816

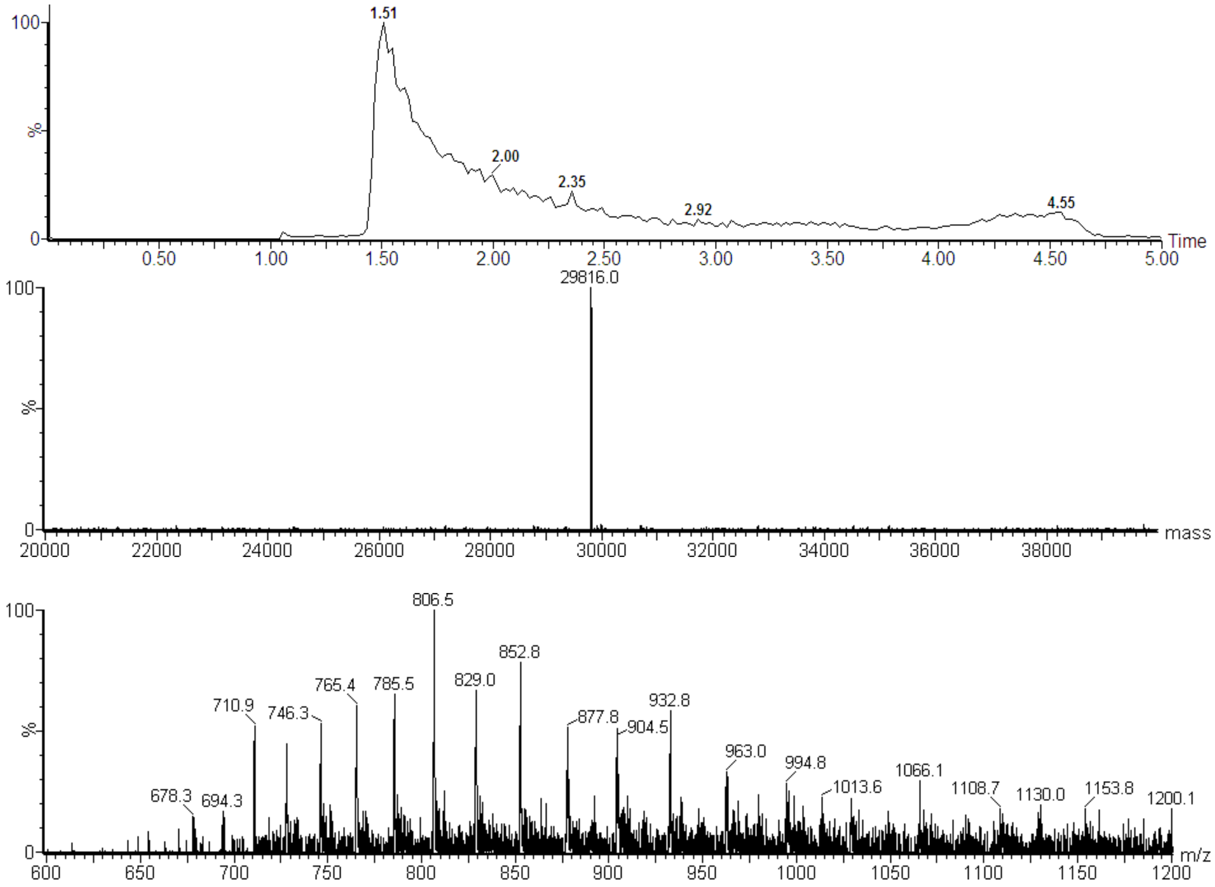

General Procedure for assessment of hydrolytic stability of protein conjugates

The protein conjugates (100 μL, [protein] 1.0 mg/mL, 100 mM sodium phosphate, 150 mM NaCl, pH 8.0) were heated at 37 °C for 4 h, and analysed by LCMS.

Hydrolytic stability of the conjugate of Grb2 SH2 (L111C) 5 with bromomaleimide 1

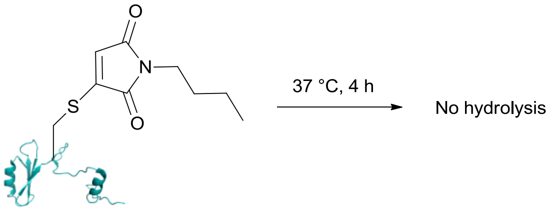

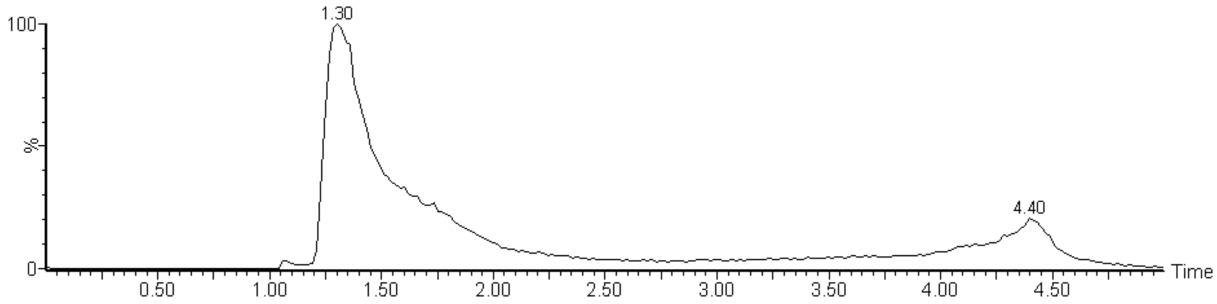

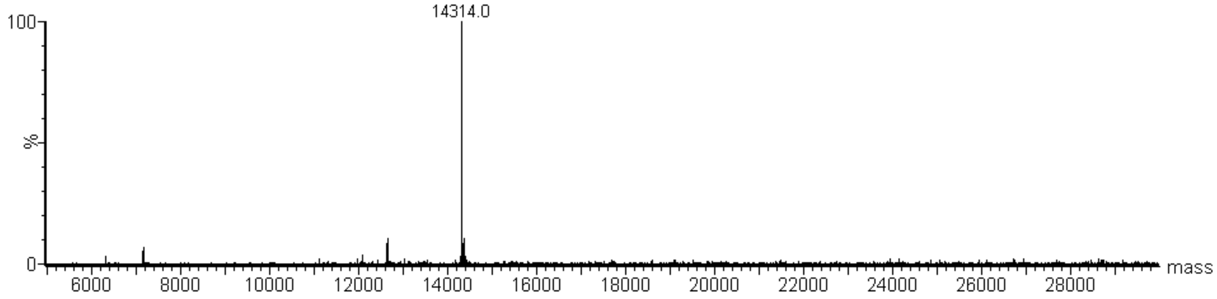

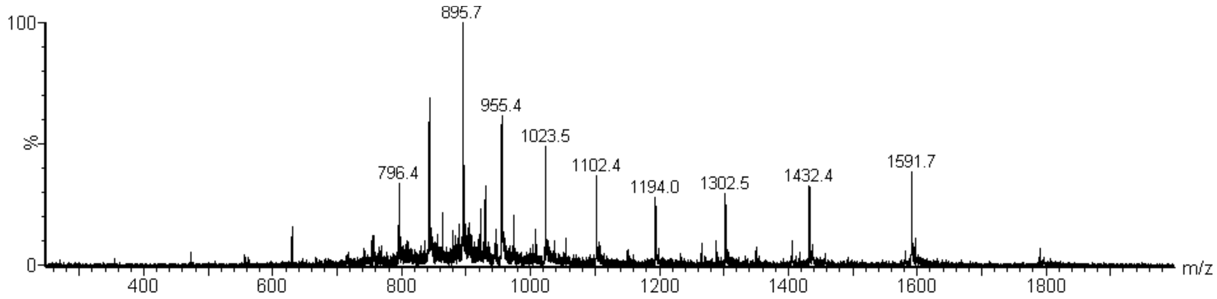

Hydrolytic stability of the conjugate of Grb2 SH2 (L111C) 5 with bromomaleimide 2

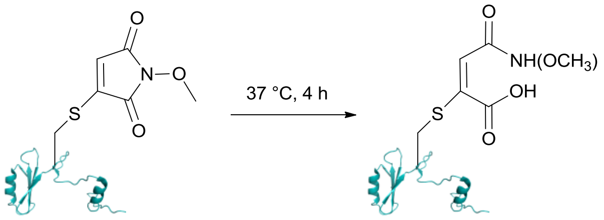

Expected Mass: 14308; Observed Mass: 14308

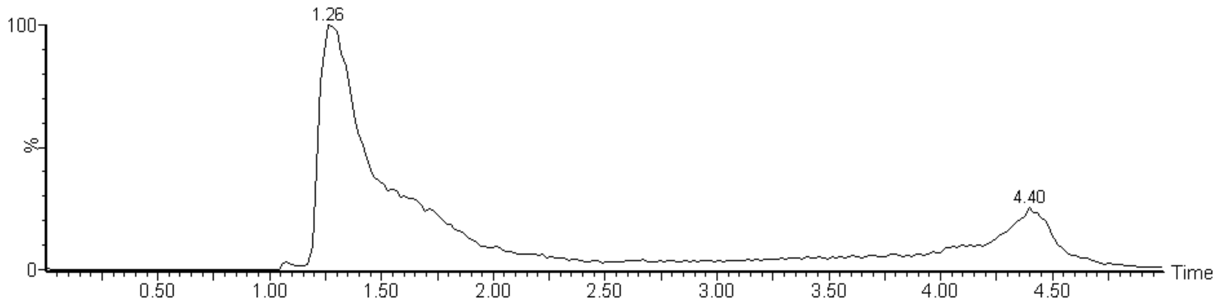

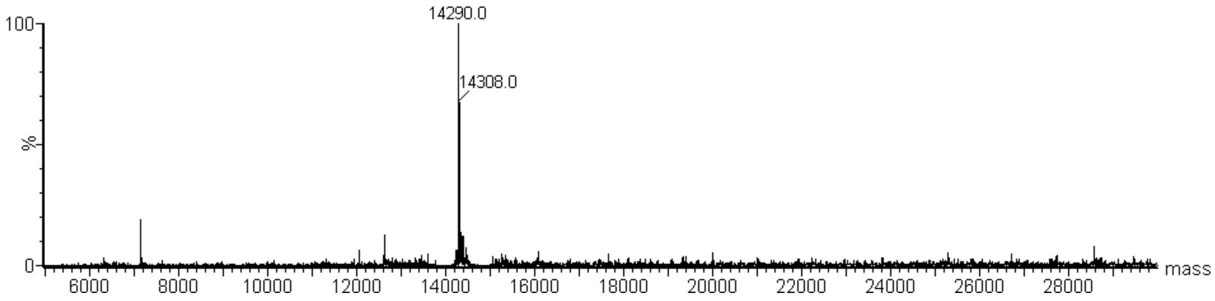

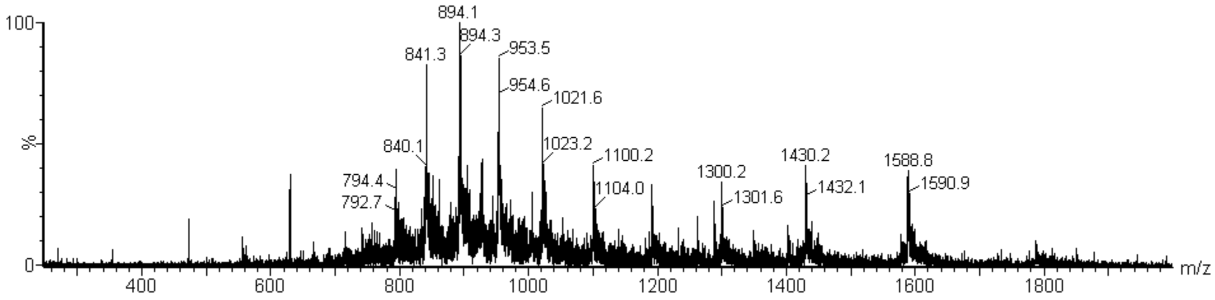

Hydrolytic stability of the conjugate of Grb2 SH2 (L111C) 5 with bromomaleimide 3

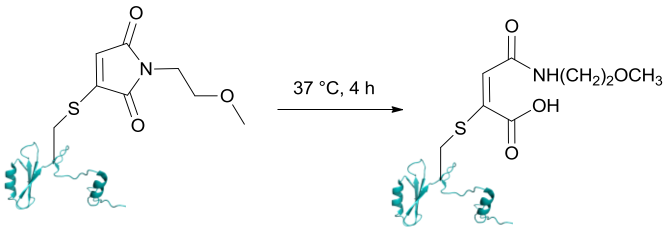

Expected Mass: 14336; Observed Mass: 14336

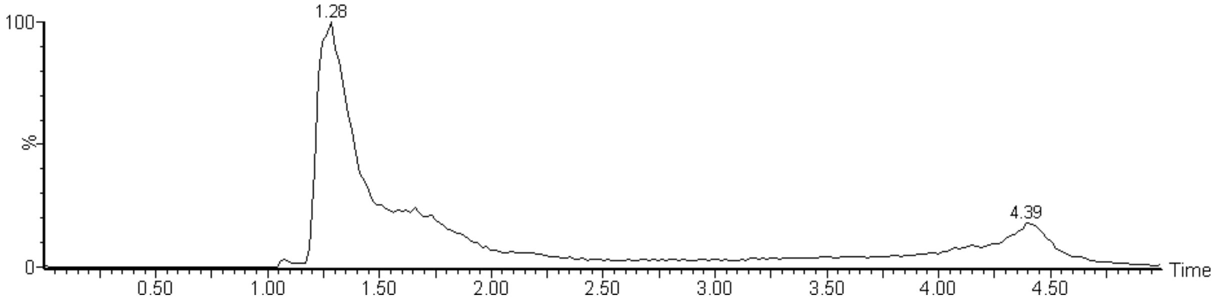

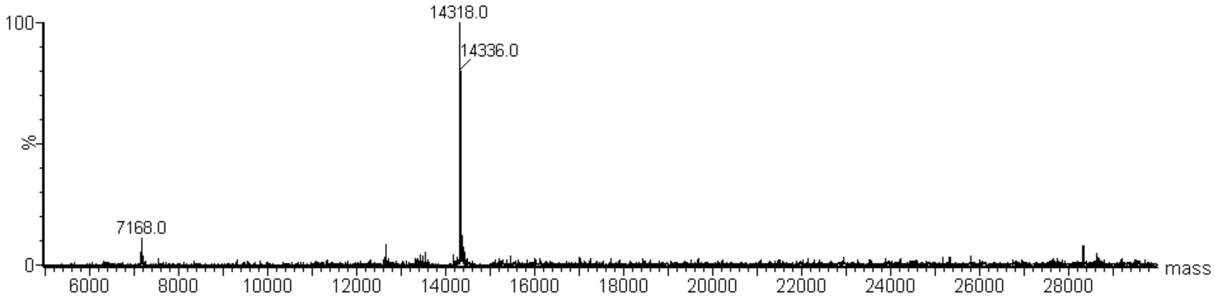

This journal is © The Royal Society of Chemistry 2013

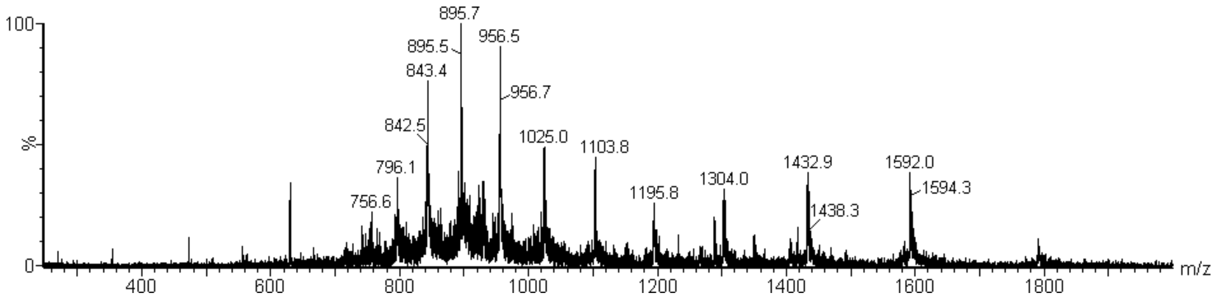

Hydrolytic stability of the conjugate of Grb2 SH2 (L111C) 5 with bromomaleimide 4

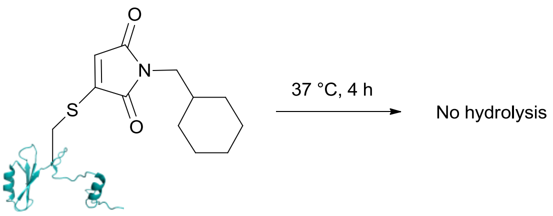

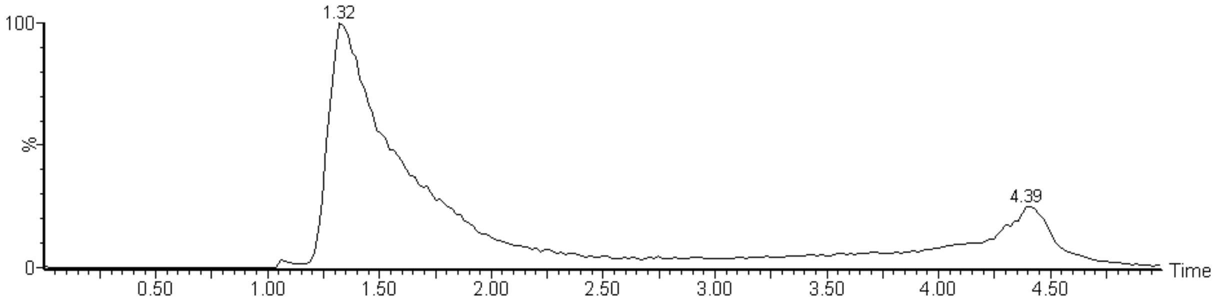

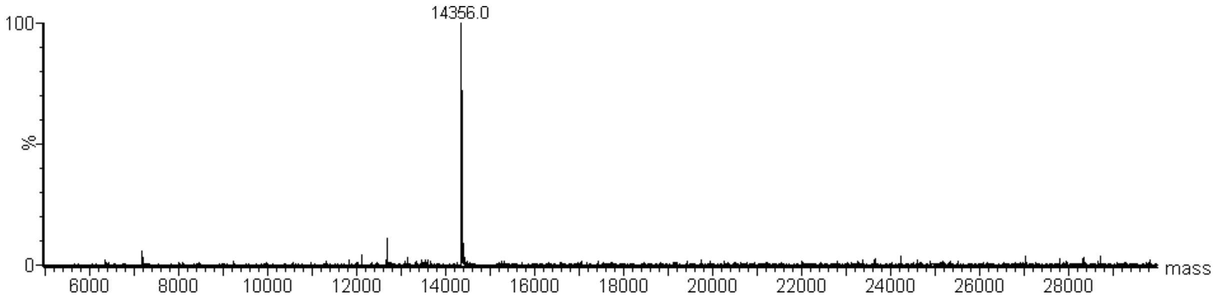

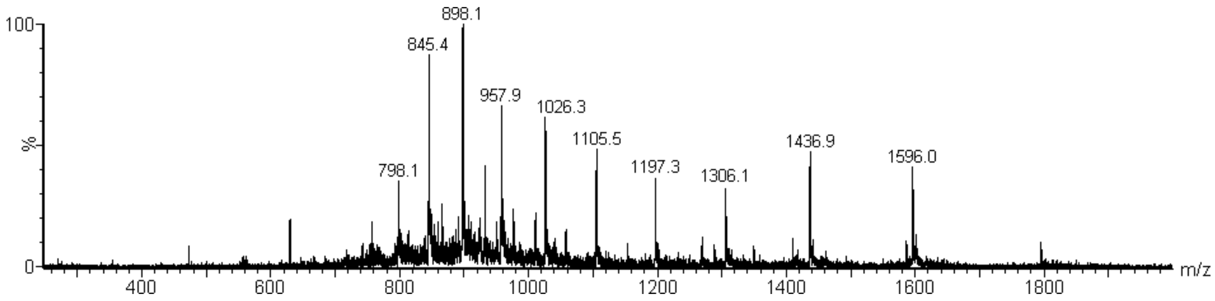

Hydrolytic stability of the conjugate of Grb2 SH2 (L111C) 5 with bromomaleimide 6

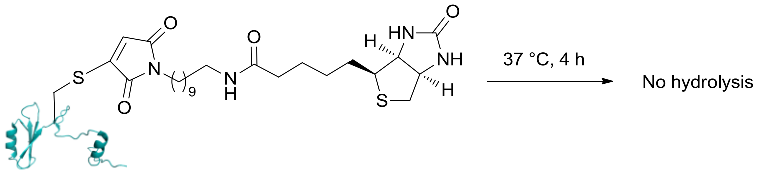

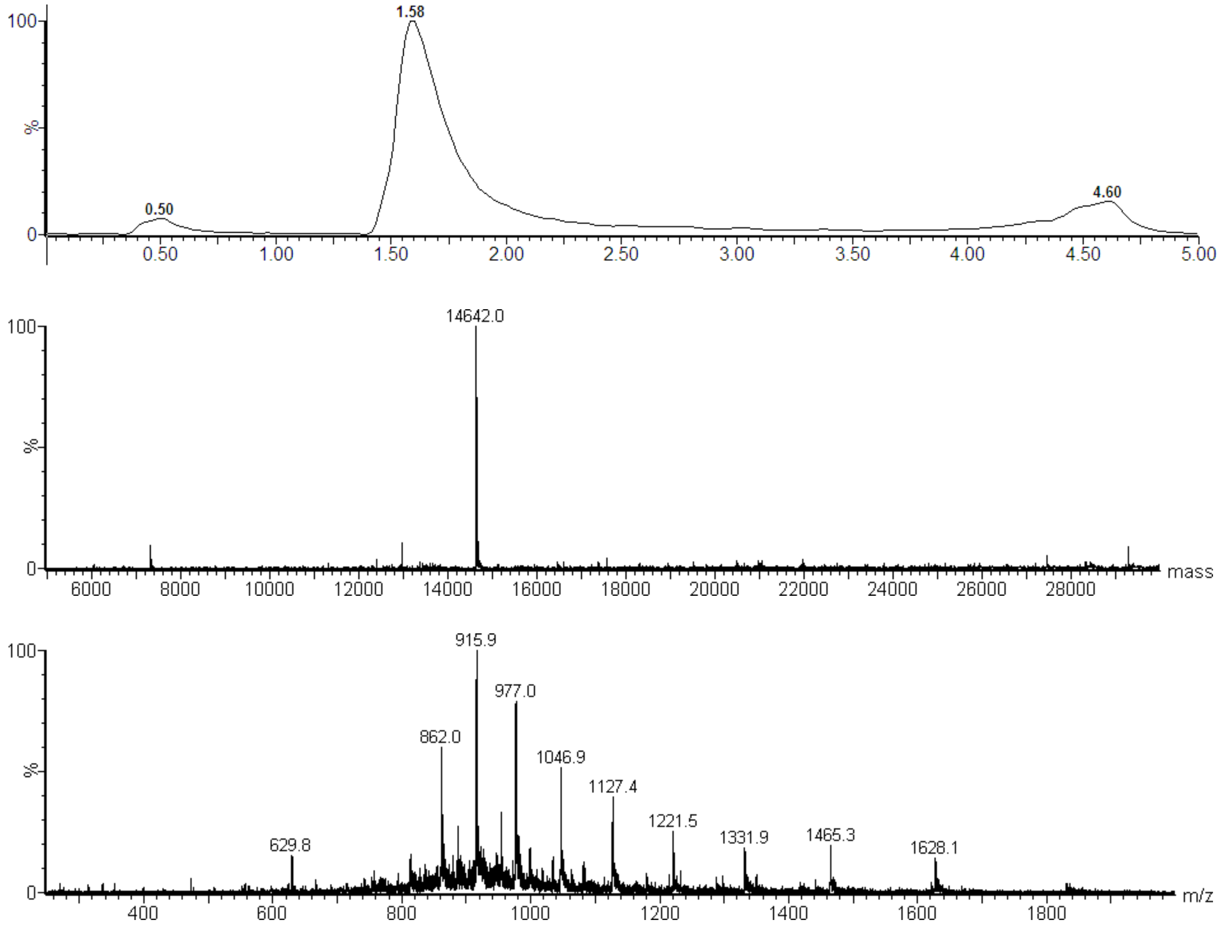

Hydrolytic stability of the conjugate of GFP (S147C) 7 with bromomaleimide 6

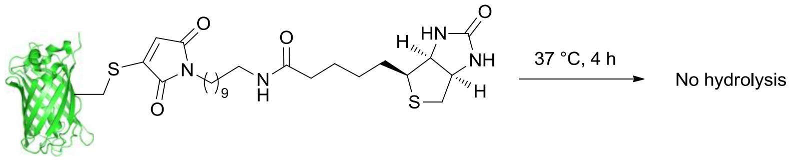

This journal is © The Royal Society of Chemistry 2013

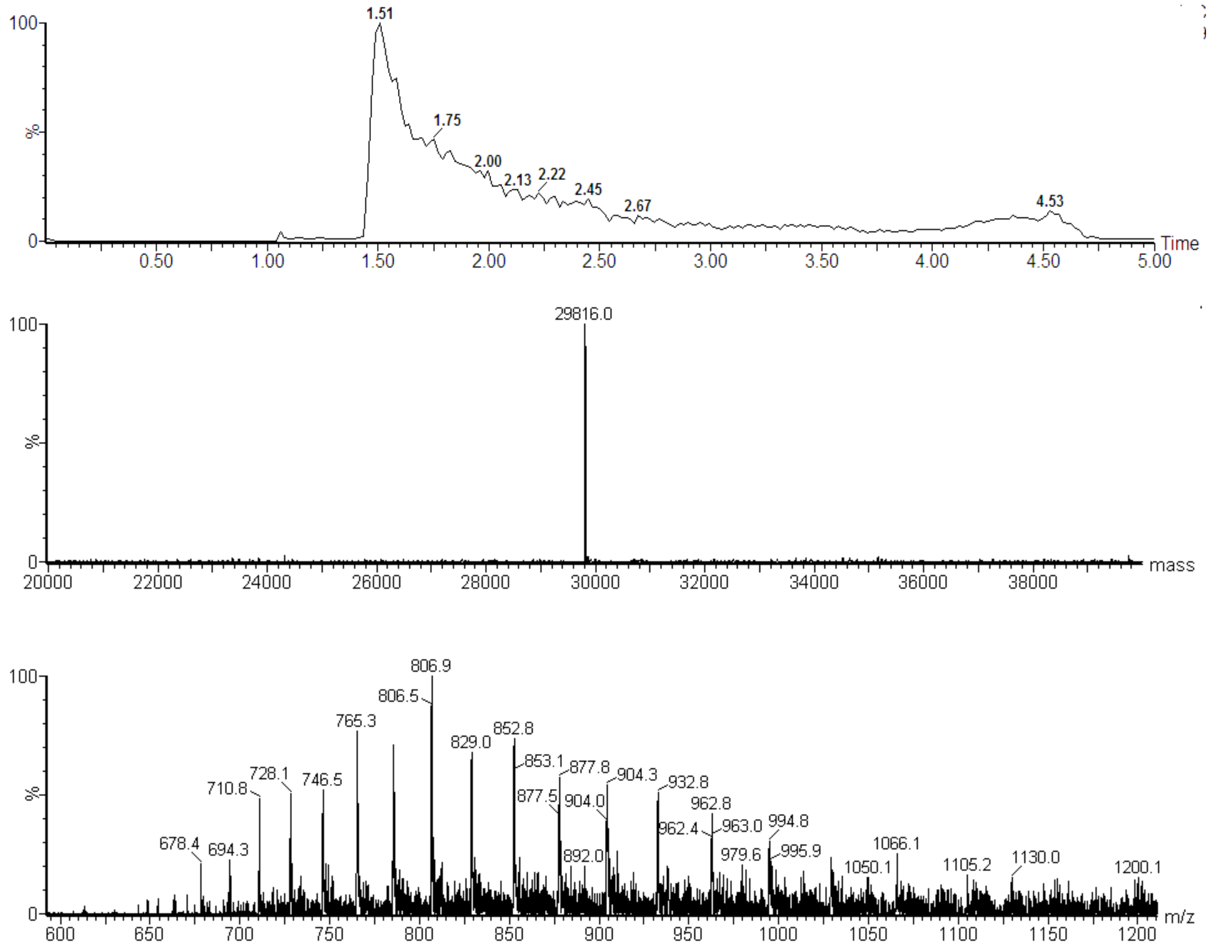

General Procedure for cleavage of protein bromomaleimide conjugates using *β*-mercaptoethanol

To a solution of protein-bromomaleimide conjugate (100 µL, [protein] 1.0 mg/mL, 100 mM sodium phosphate, 150 mM NaCl, pH 8.0) at 37 °C was added *β*-mercaptoethanol (5 µL solution in DMF, 100 equivalents). The mixture was maintained at 37 °C for 4 h and analysed by LCMS.

Cleavage of protein bromomaleimide conjugate 8 using *β*-mercaptoethanol

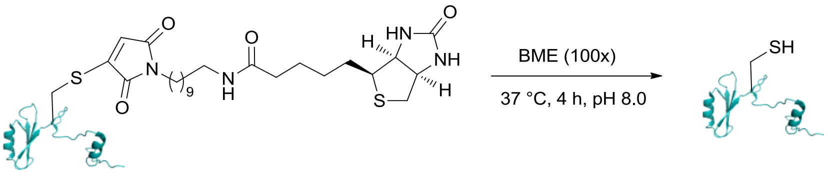

Expected Mass: 14164; Observed Mass: 14168

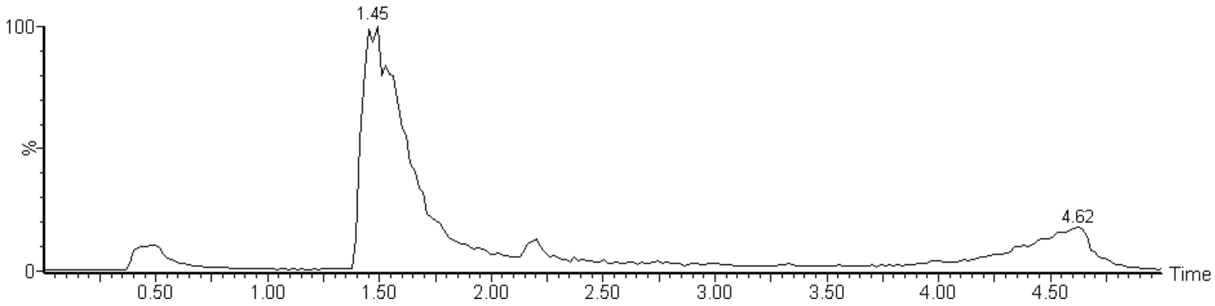

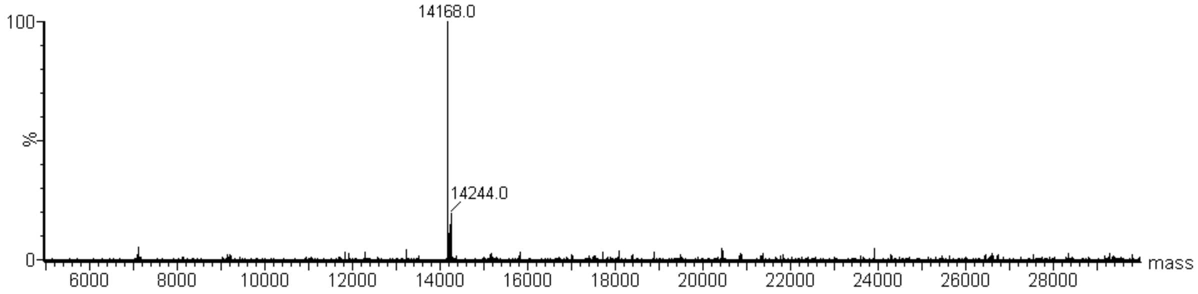

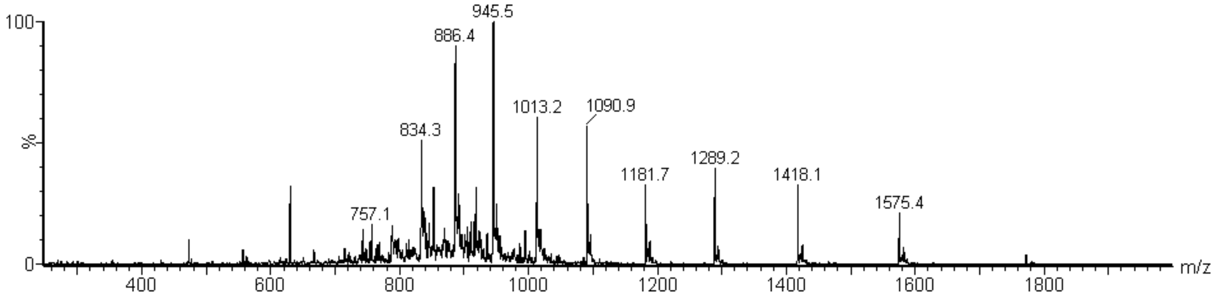

Cleavage of protein bromomaleimide conjugate 9 using *β*-mercaptoethanol

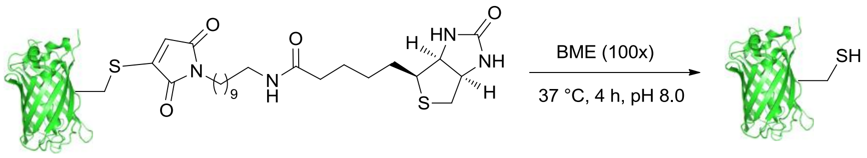

Expected Mass: 29332; Observed Mass: 29340

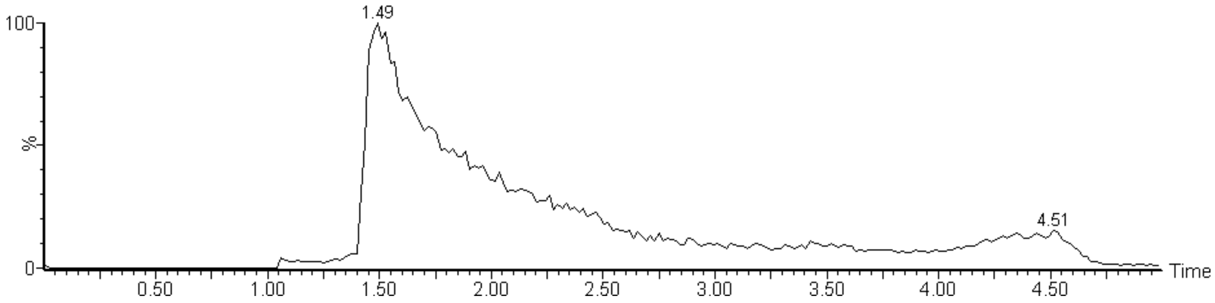

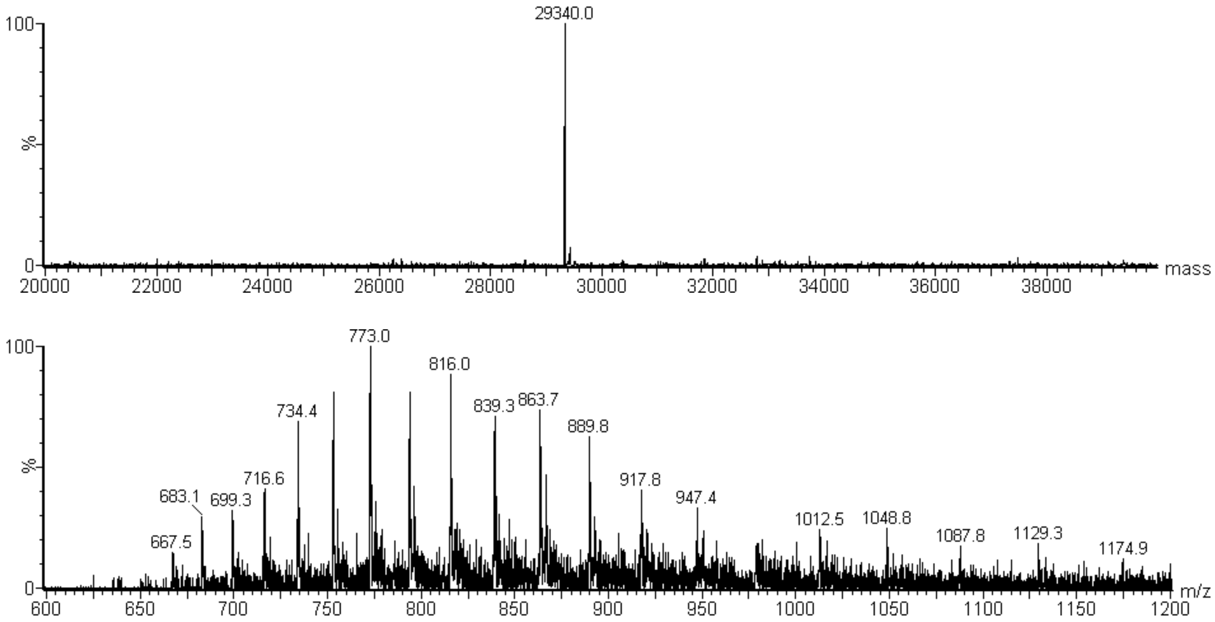

General Procedure for cleavage of protein bromomaleimide conjugates using EDT

To a solution of protein-bromomaleimide conjugate (100 μL, [protein] 1.0 mg/mL, 100 mM sodium phosphate, 150 mM NaCl, pH 8.0) at 37 °C was added ethanedithiol (5 μL solution in DMF, 1 equivalent). The mixture was maintained at 37 °C for 4 h and analysed by LCMS.

Cleavage of protein bromomaleimide conjugate 8 using EDT

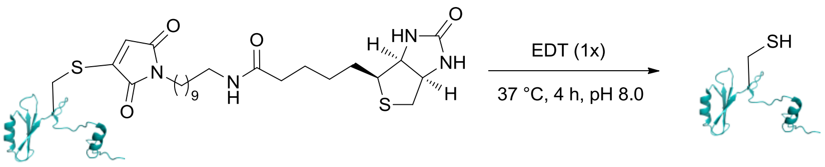

Expected Mass: 14164; Observed Mass: 14168

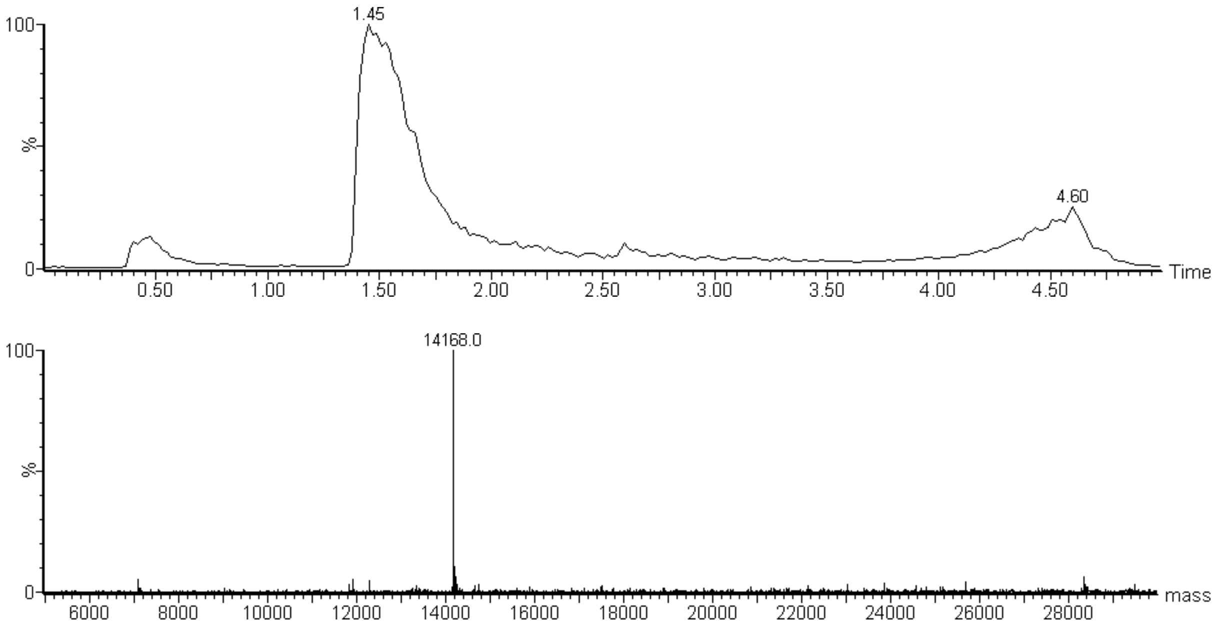

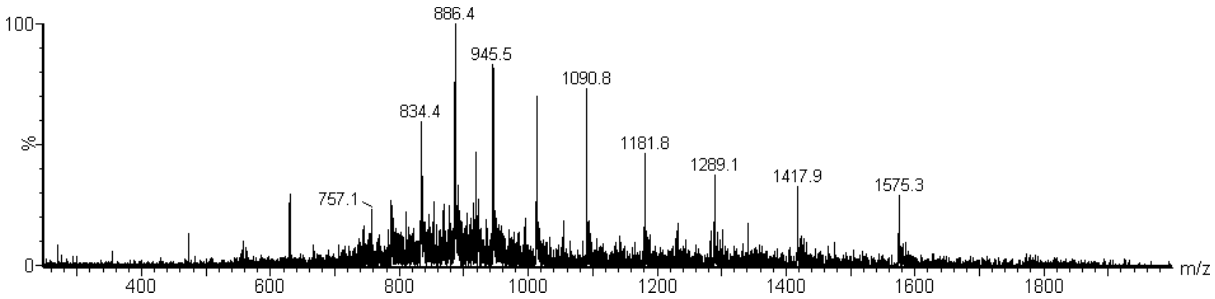

Cleavage of protein bromomaleimide conjugate 9 using EDT

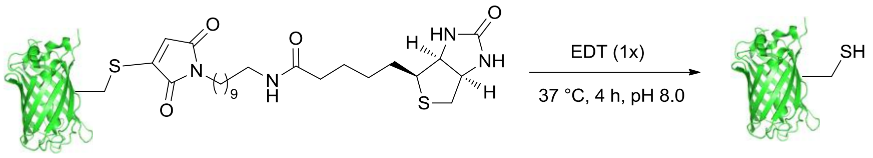

Expected Mass: 29332; Observed Mass: 29338

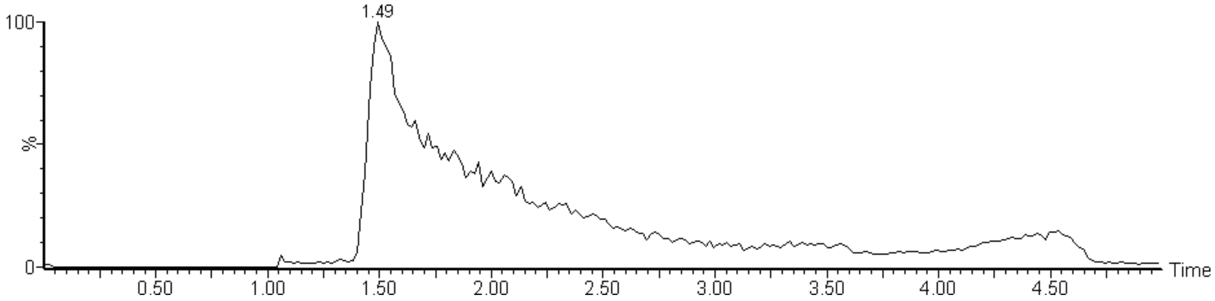

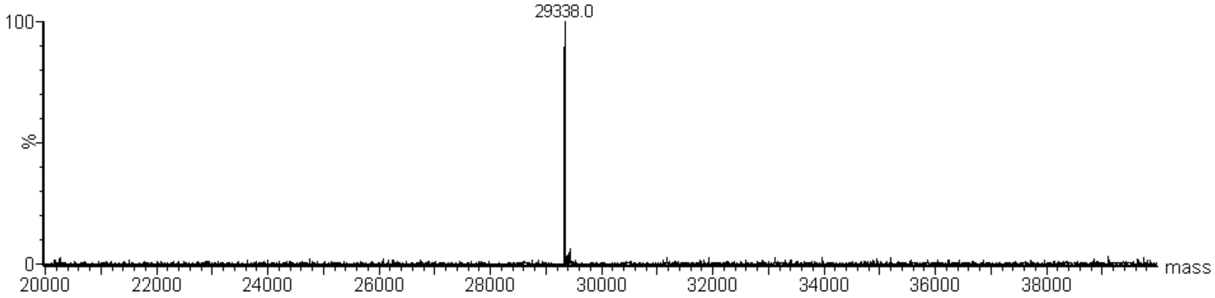

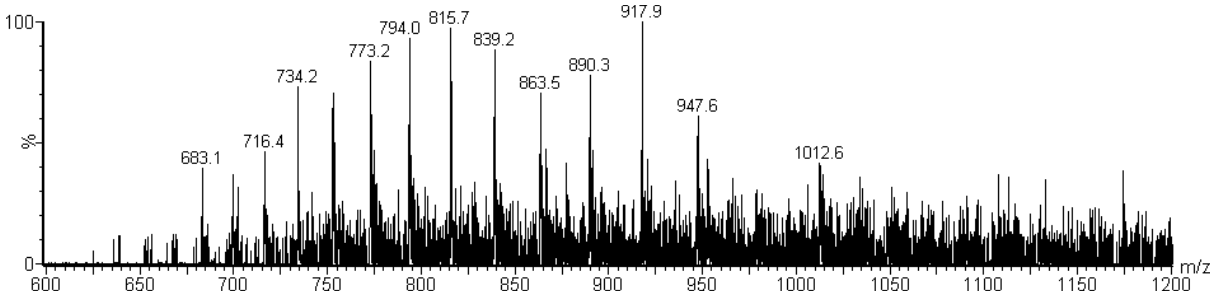

General procedure for pull down experiments with Streptavidin beads

20 μL PureProteome Streptavidin Magnetic Beads (pre-washed with 100 mM sodium phosphate, 150 mM NaCl, pH 8.0 (500 μL)) were incubated with derivatised GFP (S147C) **14** or **15** (100 μL, 1.0 mg/mL in 100 mM sodium phosphate, 150 mM NaCl, pH 8.0) with shaking (800 rpm) for 4 h at 37 °C. Following incubation, the magnetic beads were washed with 100 mM sodium phosphate, 150 mM NaCl, pH 8.0 (3 x 1 mL). 100 mM Sodium phosphate, 150 mM NaCl, pH 8.0 (100 μL) and

ethanedithiol (5 μL, 6 mM solution in DMF, 1 equivalent) were added to the beads and the mixture was shaken (800 rpm) for 4 h at 37 °C. The supernatant was removed and transferred into a Starna Scientific 26.100-F quartz fluorescence cuvette with a 10 mm path length. The fluorescence spectrum was obtained at room temperature using a Cary Eclipse Fluorescence Spectrophotometer. The sample was excited at 494 nm, and the emission intensity was scanned at 120 nm/min with an averaging time of 0.5 s and a data interval of 1 nm. The excitation and emission slit was set to 5 nm.

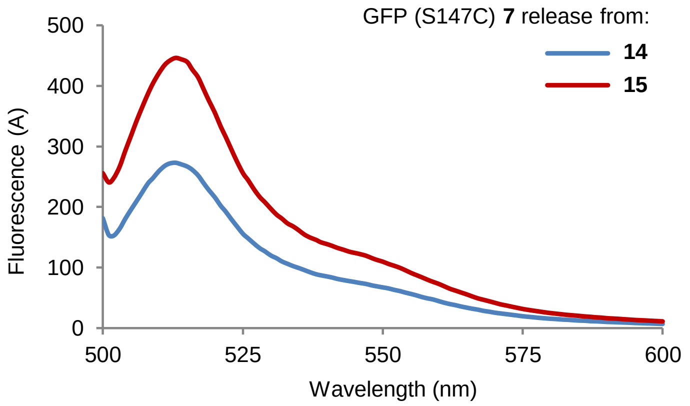

References

1. M. A. Cinelli, B. Cordero, T. S. Dexheimer, Y. Pommier and M. Cushman, *Bioorg. Med. Chem.*, 2009, 17, 7145-7155.

2. V. Chudasama, M. E. B. Smith, F. F. Schumacher, D. Papaioannou, G. Waksman, J. R. Baker and S. Caddick, *Chem. Commun.*, 2011, 47, 8781-8783.

3. R. Nathani, P. Moody, M. E. B. Smith, R. J. Fitzmaurice and S. Caddick, *ChemBioChem*, 2012, 13, 1283-1285.
